# LAMTOR5-AS1 regulates chemotherapy-induced oxidative stress by controlling the expression level and transcriptional activity of NRF2 in osteosarcoma cells

**DOI:** 10.1038/s41419-021-04413-0

**Published:** 2021-12-03

**Authors:** Youguang Pu, Yiao Tan, Chunbao Zang, Fangfang Zhao, Cifeng Cai, Lingsuo Kong, Hui Deng, Fengmei Chao, Ran Xia, Minghua Xie, Fangfang Ge, Yueyin Pan, Shanbao Cai, Dabing Huang

**Affiliations:** 1 Department of Cancer Epigenetics Program, Anhui Provincial Cancer Hospital, West Branch of the First Affiliated Hospital of USTC, Division of Life Sciences and Medicine, University of Science and Technology of China, 230001 Hefei, Anhui People’s Republic of China; 2 Department of Urology Surgery, West Branch of the First Affiliated Hospital of USTC, Division of Life Sciences and Medicine, University of Science and Technology of China, 230001 Hefei, Anhui People’s Republic of China; 3 Department of Radiation Oncology, Anhui Provincial Cancer Hospital, West Branch of the First Affiliated Hospital of USTC, Division of Life Sciences and Medicine, University of Science and Technology of China, 230001 Hefei, Anhui People’s Republic of China; 4 College of Life and Environmental Science, Wenzhou University, 325035 Wenzhou, Zhejiang People’s Republic of China; 5 Department of Anesthesiology, West Branch of the First Affiliated Hospital of USTC, Division of Life Sciences and Medicine, University of Science and Technology of China, 230001 Hefei, Anhui People’s Republic of China; 6 Department of Thoracic Tumor Surgery Department, West Branch of the First Affiliated Hospital of USTC, Division of Life Sciences and Medicine, University of Science and Technology of China, 230001 Hefei, Anhui People’s Republic of China; 7 Department of Provincial Clinical College, Wannan Medical College, 241002 Wuhu, Anhui People’s Republic of China; 8 Department of Oncology, The First Affiliated Hospital of USTC, Division of Life Sciences and Medicine, University of Science and Technology of China, 230001 Hefei, Anhui People’s Republic of China; 9 Department of Orthopedic Surgery, Anhui Provincial Cancer Hospital, West Branch of the First Affiliated Hospital of USTC, Division of Life Sciences and Medicine, University of Science and Technology of China, 230001 Hefei, Anhui People’s Republic of China

**Keywords:** Bone cancer, Cell death

## Abstract

Long-noncoding RNAs (lncRNAs) play roles in regulating cellular functions. High-throughput sequencing analysis identified a new lncRNA, termed LAMTOR5-AS1, the expression of which was much higher in the chemosensitive osteosarcoma (OS) cell line G-292 than in the chemoresistant cell line SJSA-1. Further investigations revealed that LAMTOR5-AS1 significantly inhibits the proliferation and multidrug resistance of OS cells. In vitro assays demonstrated that LAMTOR5-AS1 mediates the interaction between nuclear factor erythroid 2-related factor 2 (NFE2L2, NRF2) and kelch-like ECH-associated protein 1 (KEAP1), which regulate the oxidative stress. Further mechanistic studies revealed that LAMTOR5-AS1 inhibited the ubiquitination degradation pathway of NRF2, resulting in a higher level of NRF2 but a loss of NRF2 transcriptional activity. High level of NRF2 in return upregulated the downstream gene heme oxygenase 1 (HO-1). Moreover, NRF2 controls its own activity by promoting LAMTOR5-AS1 expression, whereas the feedback regulation is weakened in drug-resistant cells due to high antioxidant activity. Overall, we propose that LAMTOR5-AS1 globally regulates chemotherapy-induced cellular oxidative stress by controlling the expression and activity of NRF2.

## Introduction

Osteosarcoma (OS) is a common malignant tumor derived from mesenchymal tissue and commonly occurs in children and adolescents [1]. OS is highly malignant and mainly manifests with early metastasis and a low 5-year survival rate [2, 3]. Chemotherapy is a commonly used treatment method for OS and largely improves the prognosis of patients with OS [4, 5]. However, multidrug resistance usually occurs in many OS patients during chemotherapy, which leads to treatment failures, resulting in a poor prognosis of OS patients [6–8].

It was found that 98% of human genome transcripts are noncoding RNAs (ncRNAs) without a protein coding capability. Long ncRNAs (lncRNAs), greater than 200 nt in length, account for most of the ncRNA family [9]. Increasing studies have demonstrated that lncRNAs play key roles in the development and progression of human cancers, including OS [10]. A series of lncRNAs, including DANCR, SNHG12 and FOXC2-AS1, which are aberrantly expressed in OS, were proposed to regulate OS tumorigenesis and progression. However, knowledge of the functions and mechanisms of lncRNAs in OS remains limited [11–13].

Mammalian cells are often exposed to reactive oxygen species (ROS) produced by endogenous metabolism or environmental oxidants [14]. Maintaining ROS homeostasis is essential for cell survival [15]. Obviously, an ROS imbalance can lead to the development of various diseases, including cancer [16]. High ROS levels promote the growth of cells and the occurrence of tumors. In addition, excessive ROS production will lead to cell death once the toxicity threshold has been exceeded [17]. To adapt to oxidative stress, cancer cells have developed an effective ROS antioxidant system to alleviate ROS accumulation. On the other hand, elevated ROS, which are often used in conventional cancer therapies, might induce the antioxidant mechanism of tumor cells [18]. Therefore, understanding the molecular mechanism of antioxidation in tumor cells and controlling the ROS balance are needed for the treatment of malignant tumors [19, 20].

The transcription factor nuclear erythroid factor 2-like 2 (NRF2) has been recognized as a central hub that neutralizes ROS and restores the cellular redox balance [21–23]. Moreover, the (NRF2)-Kelch-like ECH-associated protein 1 (KEAP1) system, inherited from ancestors as an anti-ROS stress mechanism, is a defense system that preserves cellular homeostasis [22, 24]. The system is regulated by interactions between NRF2 and the cytosolic repressor protein KEAP1 [25].

In this study, we used high-throughput sequencing to identify a series of lncRNAs with aberrant expression in G-292 and SJSA-1 cells. We found that the lncRNA LAMTOR5-AS1 is upregulated in G-292 cells. Functional analyses revealed that LAMTOR5-AS1 inhibits OS cell proliferation and multidrug resistance. Mechanistic studies revealed that LAMTOR5-AS1 inhibits OS cell proliferation and multidrug resistance by promoting the interaction of NRF2 and KEAP1. Moreover, LAMTOR5-AS1 inhibited the ubiquitination degradation pathway of NRF2, resulting in a higher level but silenced activity of NRF2. Our findings reveal the new regulatory roles of LAMTOR5-AS1 in OS multidrug resistance mediated by the NRF2-KEAP1 system, suggesting that LAMTOR5-AS1 could be a potential new therapeutic target for OS.

## Results

### LAMTOR5-AS1 is upregulated in chemosensitive cells

**Fig. 1:**
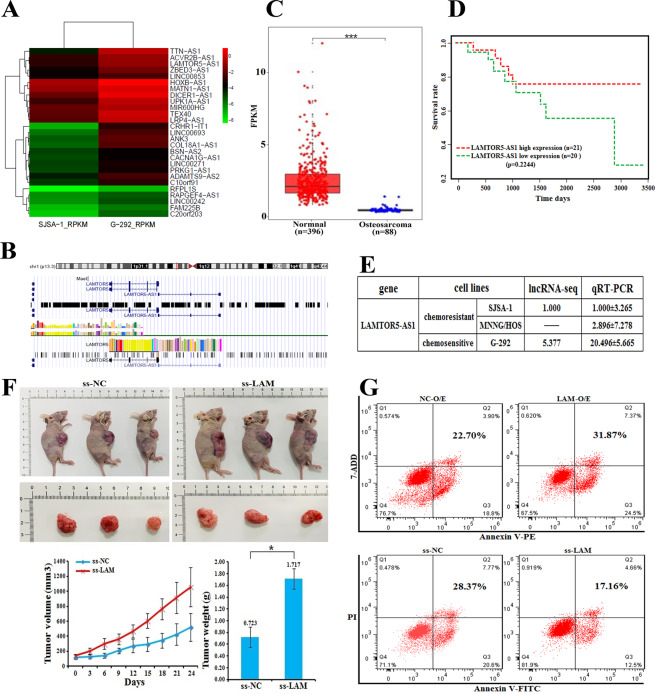
LAMTOR5-AS1 is upregulated in chemosensitive cells. **A** Hierarchical clustering analysis of RNA-seq lncRNAs that were differentially expressed between G-292 and SJSA-1 cells; the coordinates on the right represent the expression of lg(fpkm + 1). **B** LAMTOR5-AS1 is located next to the LAMTOR5 gene on human chromosome 1. LAMTOR5-AS1 is encoded by the (**+**) DNA strand, and LAMTOR5 is also coded by the (−) DNA strand. **C** Relative expression levels calculated from the LAMTOR5-AS1 FPKM data in 88 paired OS cancer samples and 396 paired surrounding normal tissues. ***p-value < 0.001. **D** Kaplan–Meier survival analysis of patient aged from 10–20 years overall survival according to LAMTOR5-AS1 levels in OS tissues. *p*-value is 0.2244. **E** The relative LAMTOR5-AS1 levels (fold changes) in chemoresistant SJSA-1 and MNNG/HOS cells versus chemosensitive G-292 cells measured by both lncRNA-seq and real-time PCR analyses are shown, “-” indicates no detection in the omics analysis. **F** Representative image of subcutaneous xenograft tumor in nude mice with smart silencer of LAMTOR5-AS1 (ss-LAM)-transfected G-292 cells compared with their control groups; the volume and weight of the tumor were also analyzed. *p-value < 0.05. **G** The effects of the forced reversal of LAMTOR5-AS1 level on the apoptosis of overexpressed of LAMTOR5-AS1 (LAM-OE) in SJSA-1 cells or the smart silencer of LAMTOR5-AS1 (ss-LAM) transfected in G-292 cells compared with their respective control groups by FACS analysis in the plot and in the original with a graph of the analyzed data and plots of the original FACS data.

LncRNA expression profiles in SJSA-1 and G-292 cells were analyzed to screen the differentially expressed lncRNAs in chemoresistant and chemosensitive cells [26]. Of the ~33,000 human lncRNAs, 1130 members exhibited the significantly differential expression, with 664 genes upregulated and 466 genes downregulated in SJSA-1 cells compared to those in G-292 cells (fold-change > 2, *p* < 0.05). Of the 1130 significantly dysregulated genes, unsupervised hierarchical clustering identified 27 lncRNAs that could distinguish G-292 cells from SJSA-1 cells (Fig. 1A, SI Appendix, Table S1). Among the 27 overexpressed lncRNAs in G-292 cells, LAMTOR5-AS1 is one of the most differentially expressed members. Moreover, qPCR confirmed five upregulated candidates, and only LAMTOR5-AS1 knockdown resulted in significant drug resistance. Thus, we focused on LAMTOR5-AS1 for further studies.

LAMTOR5-AS1 is encoded by three exons at chromosome 1 (p13.3), overlapping with the LAMTOR5 coding gene (Fig. 1B, UCSC Genome Browser). Analysis of TCGA database showed that the abundance of LAMTOR5-AS1 was lower in OS tissue than in normal muscle tissue. Higher expression of LAMTOR5-AS1 correlates with a better prognosis for OS patients aged 10–20 years (Fig. 1C, D). The expression of LAMTOR5-AS1 in G-292, SJSA-1 and MNNG/HOS cells was consistent with the results of high-throughput sequencing. LAMTOR5-AS1 has a highest expression in G-292 cells in contrast to the lowest expression in SJSA-1 cells (Fig. 1E). More interestingly, we found that LAMTOR5-AS1 was closely related to the apoptosis of OS cells. Downregulation of LAMTOR5-AS1 inhibited the apoptosis of G-292 cells in vivo (Fig. 1F) and in vitro (Fig. 1G), and the expression of apoptosis related proteins in frozen tumor tissues is consistent (SI Appendix, Fig. S1), whereas the opposite effect was found when LAMTOR5-AS1 was overexpression of SJSA-1 cells in vivo (Fig. 1G).

### LAMTOR5-AS1 expression inhibits the drug resistance of OS cells

**Fig. 2:**
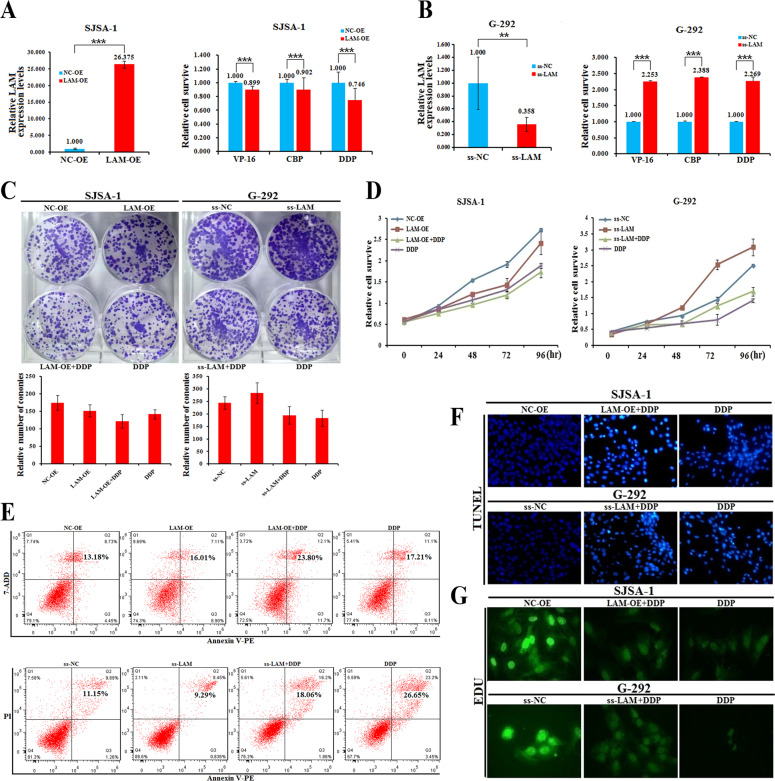
LAMTOR5-AS1 expression inhibits the drug resistance of OS cells. **A** The relative LAMTOR5-AS1 expression level (fold-change) in SJSA-1 cells transfected with LAMTOR5-AS1-overexpressing lentivirus (LAM-OE) versus the negative control (NC-OE). CCK-8 assays showing cell death triggered by the IC_50_ dose of drugs in SJSA-1 cells transfected with LAMTOR5-AS1-overexpressing lentivirus (LAM-OE) versus the negative control (NC-OE). ***p-value < 0.001. **B** The relative LAMTOR5-AS1 expression level (fold) in G-292 cells transfected with the smart silencer of LAMTOR5-AS1 (ss-LAM) versus the negative control (ss-NC). CCK-8 assays showing cell death triggered by the IC_50_ dose of drugs in G-292 cells transfected with the smart silencer of LAMTOR5-AS1 (ss-LAM) versus the negative control (ss-NC). n.s, no statistical significance; ***p-value < 0.001. **C** Colony formation assays showed that LAMTOR5-AS1 inhibited cell proliferation in SJSA-1 cells treated with LAMTOR5-AS1-overexpressing lentivirus (LAM-OE), overexpression lentivirus with DDP (LAM-OE + DDP) or DDP alone versus the negative control (NC-OE), and G-292 cells treated with the smart silencer of LAMTOR5-AS1 (ss-LAM), the smart silencer of LAMTOR5-AS1 with DDP (ss-LAM + DDP) or DDP alone versus the negative control (ss-NC). **D** CCK-8 assays every 24 h showed that LAMTOR5-AS1 inhibited proliferation of SJSA-1 cells treated with LAMTOR5-AS1-overexpressing lentivirus (LAM-OE), overexpression lentivirus with DDP (LAM-OE + DDP) or DDP alone versus the negative control (NC-OE), and G-292 cells treated with the smart silencer of LAMTOR5-AS1 (ss-LAM), the smart silencer of LAMTOR5-AS1 with DDP (ss-LAM + DDP) or DDP alone versus the negative control (ss-NC). **E** LAMTOR5-AS1 induced apoptosis as shown in annexin V-FITC staining assays in SJSA-1 cells treated with LAMTOR5-AS1-overexpressing lentivirus (LAM-OE), overexpression lentivirus with DDP (LAM-OE + DDP) or DDP alone versus the negative control (NC-OE), and G-292 cells treated with the smart silencer of LAMTOR5-AS1 (ss-LAM), the smart silencer of LAMTOR5-AS1 with DDP (ss-LAM + DDP) or DDP alone versus the negative control (ss-NC). **F** LAMTOR5-AS1 induced apoptosis as shown in TUNEL staining assays in SJSA-1 cells treated with LAMTOR5-AS1-overexpressing lentivirus (LAM-OE), overexpression lentivirus with DDP (LAM-OE + DDP) or DDP alone versus the negative control (NC-OE), and G-292 cells treated with the smart silencer of LAMTOR5-AS1 (ss-LAM), the smart silencer of LAMTOR5-AS1 with DDP (ss-LAM + DDP) or DDP alone versus the negative control (ss-NC). **G** LAMTOR5-AS1 inhibited cell proliferation as shown in EdU staining assays in SJSA-1 cells treated with LAMTOR5-AS1-overexpressing lentivirus (LAM-OE), overexpression lentivirus with DDP (LAM-OE + DDP) or DDP alone versus the negative control (NC-OE), and G-292 cells treated with the smart silencer of LAMTOR5-AS1 (ss-LAM), the smart silencer of LAMTOR5-AS1 with DDP (ss-LAM + DDP) or DDP alone versus the negative control (ss-NC).

Previous studies have found that LAMTOR5-AS1 is downregulated in the drug-resistant cell line SJSA-1, whereas it is highly expressed in the drug-sensitive cell line G-292. To further investigate the role of LAMTOR5-AS1 on the drug resistance of OS cells, we overexpressed or downregulated LAMTOR5-AS1 in SJSA-1 or G-292 cells (Fig. 2A, B). Drug resistance assays showed that overexpression of LAMTOR5-AS1 could significantly inhibit the survival of drug-resistant SJSA-1 cells in the presence of the drugs VP-16 (Etoposide), CBP (Carboplatin) and DDP (Cisplatin), while downregulation of LAMTOR5-AS1 promoted the tolerance of drug-sensitive G-292 cells in the presence of VP-16, CBP and DDP (Fig. 2A, B). Moreover, the colony formation and cell proliferation assays showed that overexpression of LAMTOR5-AS1 significantly inhibited the proliferation of SJSA-1 cells by DDP treatment, whereas downregulation of LAMTOR5-AS1 enhanced the survival and proliferation of G-292 cells by DDP treatment (Fig. 2C, D). Similarly, we also found that overexpression of LAMTOR5-AS1 promoted DDP-induced apoptosis of SJSA-1 cells, as detected by apoptosis, TUNEL and EdU assays, while downregulation of LAMTOR5-AS1 inhibited the apoptosis of G-292 cells (Fig. 2E–G).

### NRF2 is induced by DDP and positively correlates with OS drug resistance

**Fig. 3:**
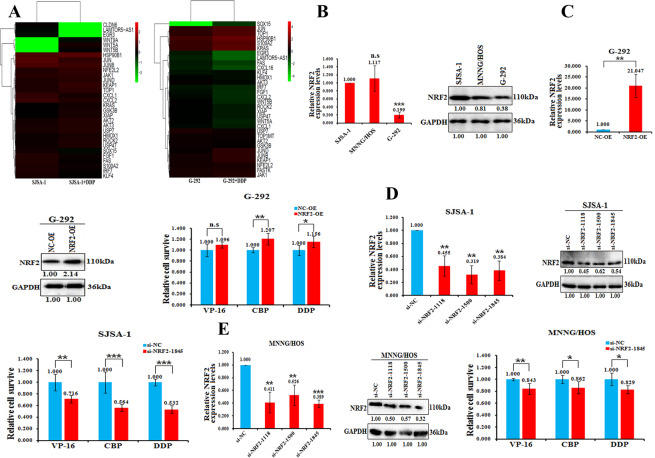
NRF2 is induced by DDP and positively correlates with OS drug resistance. **A** Heatmap analysis of differentially expressed genes with polyA-seq in SJSA-1 and G-292 cells versus the control treated with the IC_50_ dose of DDP. **B** Levels of NRF2 in chemoresistant SJSA-1 and MNNG/HOS cells versus chemosensitive G-292 cells measured by real-time PCR and western blot analyses are shown. n.s, no statistical significance; ***p-value < 0.001. **C** The levels of NRF2 in G-292 cells transfected with NRF2-OE versus the negative control (NC-OE) measured by real-time PCR and western blot analyses are shown. CCK-8 assays showing cell death triggered 72 h by the IC_50_ dose of three drugs in G-292 cells transfected with NRF2-OE versus the negative control (NC-OE). n.s, no statistical significance; *p-value < 0.05; **p-value < 0.01. **D** The mRNA and protein levels of NRF2 determined by real-time PCR and western blot analyses in the three different regions of siRNAs transfected into SJSA-1 cells versus the negative control (si-NC). The CCK-8 assays showing cell death triggered 72 h by the IC_50_ dose of three drugs in SJSA-1 cells transfected with the three different regions of siRNAs versus the negative control (si-NC). **p-value < 0.01; ***, *p*-value < 0.001. **E** The mRNA and protein levels of NRF2 determined by real-time PCR and western blot analyses in the three different regions of siRNAs transfected into MNNG/HOS cells versus the negative control (si-NC). The CCK-8 assays showing cell death triggered 72 h by the IC_50_ dose of three drugs in MNNG/HOS cells transfected with the three different regions of siRNAs versus the negative control (si-NC). *p-value < 0.05; **p-value < 0.01; ***p-value < 0.001.

Next, we determined the mRNA expression profiles of SJSA-1 and G-292 cells induced by DDP and screened the differentially expressed mRNAs. Of the ~11,000 human mRNAs analyzed, 31 genes showed the same expression trend in SJSA-1 and G-292 cells (Fig. 3A). One of the members, NRF2 is a receptor for exogenous toxic substances and oxidative stress and plays an important role in the main defense mechanism involved in cell antioxidative stress and the induction of exogenous toxic substances [27]. In addition, NRF2 also involves in the drug resistance and survival of cancer cells [28]. Notably, NRF2 was predicted to bind to the promoter region of LAMTOR5-AS1 (http://jaspar.genereg.net). Thus, we analyzed the expression of NRF2 in SJSA-1, MNNG/HOS and G-292 cells. The results demonstrated that NRF2 showed the highest expression in SJSA-1 cells and the lowest expression in G-292 cells at both mRNA and protein levels, which was consistent with the drug resistance phenotypes of SJSA-1 and G-292 cells (Fig. 3B).

Moreover, we investigated the role of the NRF2 gene on the drug resistance of OS cells by overexpressing or downregulating its expression. Overexpression of NRF2 significantly promoted the resistance of G-292 to CBP and DDP except VP-16 (Fig. 3C), while downregulating NRF2 expression with three independent NRF2-specific siRNAs transduced into SJSA-1 and MNNG/HOS cells had the opposite effect (Fig. 3D, E). These results indicate that DDP-induced NRF2 expression is closely related to the drug resistance of OS cells.

### NRF2 directly regulates the expression of LAMTOR5-AS1

**Fig. 4:**
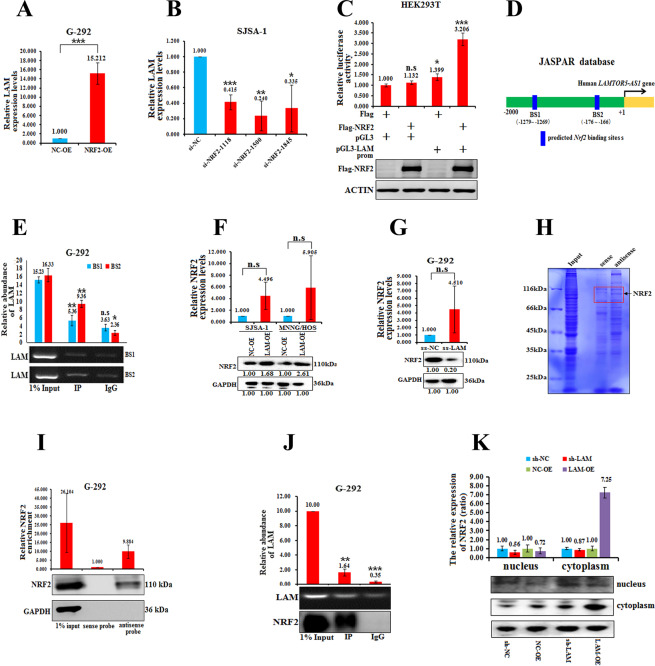
NRF2 directly regulates the expression of LAMTOR5-AS1, which feedback regulates NRF2 protein expression. **A** The levels of LAMTOR5-AS1 in G-292 cells transfected with NRF2-OE versus the negative control (NC-OE) measured by real-time PCR analyses. ***p-value < 0.001. **B** The levels of LAMTOR5-AS1 measured by real-time PCR analyses in the NRF2 three different regions of siRNAs transfected into SJSA-1 cells versus the negative control (si-NC), respectively. *p-value < 0.05; **p-value < 0.01; ***p-value < 0.001. **C** LAMTOR5-AS1 promoter was obtained by whole-gene synthesis and constructed into pGL3-basic vector, the constructed vector was cotransfected with the NRF2-overexpressing plasmid 3xFlag-NRF2, and a luciferase reporter gene assay was used to detect the binding of NRF2 to LAMTOR5-AS1. n.s, no statistical significance; *p-value < 0.05; ***p-value < 0.001. **D** Two potential NRF2-binding sites in the LAMTOR5-AS1 promoter region were predicted in the high-quality transcription factor-binding profile database (JASPAR). **E** Further verify the combination of the two potential NRF2-binding sites in the LAMTOR5-AS1 promoter region with ChIP-qPCR experiments. n.s, no statistical significance; **p-value < 0.01; ***p-value < 0.001. **F** The levels of NRF2 in SJSA-1 and MNNG/HOS cells transfected with LAMTOR5-AS1-overexpressing lentivirus (LAM-OE) versus the negative control (NC-OE) measured by real-time PCR and western blot analyses. n.s, no statistical significance. **G** The levels of NRF2 with the smart silencer of LAMTOR5-AS1 (ss-LAM) transfected in G-292 cells versus the negative control (ss-NC) measured by real-time PCR and western blot analyses. n.s, no statistical significance. **H** Visualization of protein bands stained with Coomassie brilliant blue pulled down by biotin-labeled antisense probes against LAMTOR5-AS1 in total protein extracts of G-292 cells. Protein identities with high probabilities as determined using mass spectrometry are labeled. **I** HEK293T cells were transfected with biotinylated LAMTOR5-AS1 sense probe and antisense probe 20 μl streptavidin beads were washed once with RPD buffer, then 3 μg of sense probe (each 1 μg of 1, 2, 3 probes) and 3 μg of antisense probe (each 1 μg of 1, 2, 3 probes) were added, respectively, and incubated for 3 h at 4 °C). After transfection for 48 h, cells were collected for the biotin-based pull-down assay. NRF2 expression levels were analyzed by western blotting. *p-value < 0.05; **p-value < 0.01. **J** RIP assays using an anti-NRF2 antibody showed that NRF2 interacts with LAMTOR5-AS1 in G-292 cells. The expression of LAMTOR5-AS1 analyzed by real-time PCR results of RIP assays are shown in the top. The results of agarose electrophoresis of the PCR products are shown in the middle. The NRF2 levels were also analyzed by western blots in the bottom. **p-value < 0.01; ***p-value < 0.001. **K** Cell transfected with LAM-OE or ss-LAM. Then fractions were isolated from cytoplasmatic and nuclear, and the expression of NRF2 were measured by western blot.

We investigated the relationship between LAMTOR5-AS1 and NRF2 by overexpressing and downregulating NRF2 in G-292 and SJSA-1 OS cells, respectively. Overexpression of NRF2 significantly increased the expression of LAMTOR5-AS1, while downregulation of NRF2 significantly inhibited the expression of LAMTOR5-AS1 (Fig. 4A, B). Dual-luciferase reporter assays were performed to test these interactions. Transfection of the pCDNA3.1-NRF2 plasmid into HEK293T cells promoted the luciferase activity of pGL3-LAMTOR5-AS1-promoter-luc (Fig. 4C). Further sequence analysis predicted two NRF2-binding motifs(NRF2-BS1:-1,279 to -1,269 bp and NRF2-BS2:-176 to -166bp) in the LAMTOR5-AS1 promoter region (Fig. 4D). Further ChIP-qPCR confirmed that NRF2 bound to the BS2 region of LAMTOR5-AS1 promoter is more specific (Fig. 4E). These results indicate that NRF2 can directly bind to BS2 region of LAMTOR5-AS1 promoter to promote its transcription

### LAMTOR5-AS1 feedback regulates NRF2 expression at the protein level by increasing its stability

Notably, overexpression of LAMTOR5-AS1 in SJSA-1 and MNNG/HOS cells increased NRF2 expression at both mRNA and protein levels, while downregulation of LAMTOR5-AS1 in G-292 cells decreased the NRF2 protein level but increased the NRF2 mRNA level (Fig. 4F, G), indicating that LAMTOR5-AS1 expression is positively correlated with NRF2 at the protein level rather than at the mRNA level. To further identify whether the NRF2 protein is directly regulated by LAMTOR5-AS1, we detected LAMTOR5-AS1 binding proteins by RNA pull-down (RPD) assays. The precipitated proteins were subjected to gel electrophoresis analysis, and several differential bands were selected for mass spectrometry analysis (Fig. 4H) [29]. Based on the functional annotation of proteins predicted by mass spectrometry analysis, NRF2 was deemed as an LAMTOR5-AS1-associated protein. Western blotting further confirmed the binding of LAMTOR5-AS1 to NRF2 (Fig. 4I). RNA immunoprecipitation (RIP) assays using an anti-NRF2 antibody also demonstrated the association between NRF2 and LAMTOR5-AS1 (Fig. 4J). Taken together, these data suggest that LAMTOR5-AS1 physically associates with NRF2. At the same time, nuclear-cytoplasmic separation assay showed the protein level and the subcellular localization of NRF2 is regulated by LAMTOR5-AS1, the expression of LAMTOR5-AS1 can inhibit the degradation of NRF2 in the cytoplasm and the entry of NRF2 into the nucleus (Fig. 4K).

**Fig. 5:**
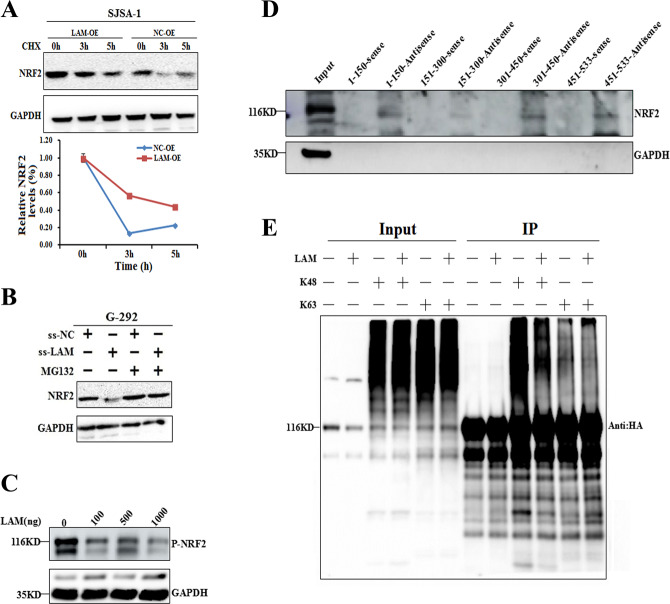
LAMTOR5-AS1 feedback regulates NRF2 protein expression by increasing its stability. **A** The protein levels of NRF2 were measured in SJSA-1 cells transfected with LAMTOR5-AS1-overexpressing lentivirus (LAM-OE) versus the negative control (NC-OE) treated with CHX (50 mg/ml) for 3 or 5 h before harvest and analyzed by western blot. **B** The protein levels of NRF2 were checked with the smart silencer of LAMTOR5-AS1 (ss-LAM) versus the negative control (ss-OE) in G-292 cells treated with MG132 (20 mmol/l) for 3 h before harvest and analyzed by western blot. **C** Different concentrations of LAMTOR5-AS1 (0, 100, 500, and 1000 ng) plasmid were transfected into the cells, the expression of phosphorylated NRF2 was detected by western blot. **D** The LAMTOR5-AS1 segments probes was designed, the binding region between LAMTOR5-AS1 and NRF2 was analyzed by RNA pull-down assay and western blot. **E** The LAMTOR5-AS1 overexpression plasmid was cotransfected with HA-UB (K48) and HA-UB (K63) plasmids, respectively. Co-IP test was performed with NRF2 antibody and western blot was performed with HA antibody. The effect of LAMTOR5-AS1 on the ubiquitination of NRF2 was analyzed.

As LAMTOR5-AS1 can increase the levels of the NRF2 protein at the posttranscriptional level, we further used the protein synthesis inhibitor cycloheximide (CHX) and the protein ubiquitin inhibitor (MG132) to evaluate the effect of LAMTOR5-AS1 on the degradation of NRF2. LAMTOR5-AS1 overexpression in OS cells prolonged the half-life of NRF2 (Fig. 5A, B). As this result further confirms that LAMTOR5-AS1 regulates NRF2 expression by affecting the half-life. However, the increase in the NRF2 protein level mediated by LAMTOR5-AS1 cannot explain its role in promoting apoptosis and inhibiting drug resistance. The phosphorylation of NRF2 can promote its ubiquitination. We further studied the mechanism of LAMTOR5-AS1 inhibiting the ubiquitination of NRF2 by transfecting different amounts of LAMTOR5-AS1 overexpression plasmids (0, 100, 500, and 1000 ng) into G-292 cells and western blot analysis showed that LAMTOR5-AS1 expression significantly inhibited NRF2 phosphorylation (Fig. 5C). More interestingly, LAMTOR5-AS1 expression also inhibited the interaction between KEAP1 and NRF2 protein (Fig. 5D). To further clarify the inhibition type of LAMTOR5-AS1 on NRF2 polyubiquitination, LAMTOR5-AS1 was cotransfected with K48-HA and K63-HA plasmids respectively and Co-IP results showed that LAMTOR5-AS1 inhibited NRF2 K48 type ubiquitination (Fig. 5E). These results indicate that LAMTOR5-AS1 can inhibit K48 polyubiquitination by inhibiting NRF2 phosphorylation and binding with KEAP1.

### LAMTOR5-AS1 inhibits NRF2 function by affecting its transcriptional activity

**Fig. 6:**
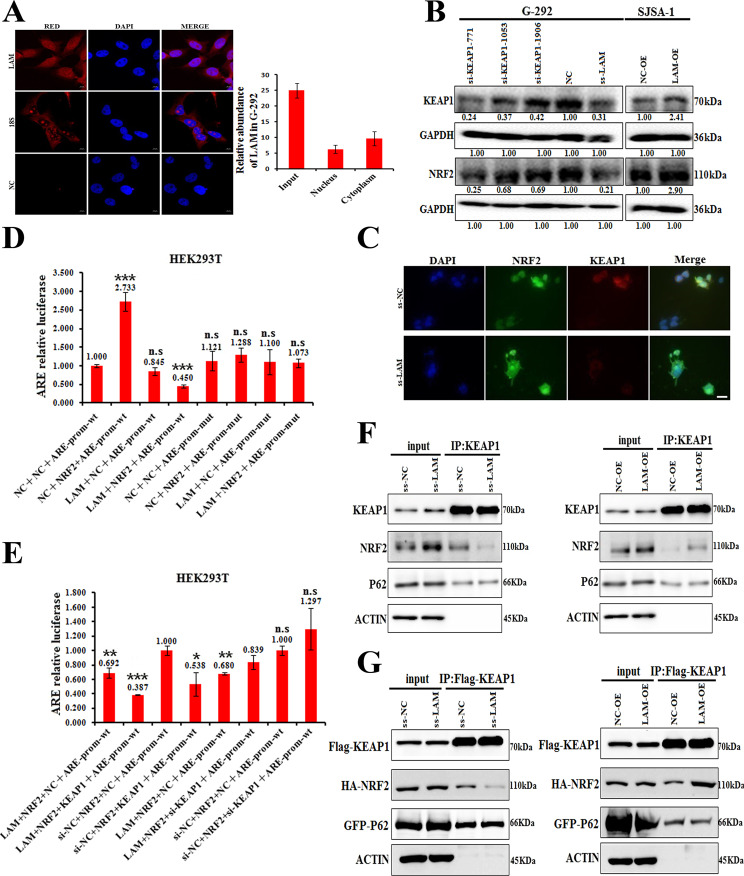
LAMTOR5-AS1 inhibits NRF2 function by affecting its transcriptional activity. **A** RNA fluorescence in situ hybridization showing the localization of LAMTOR5-AS1 in G-292 cells. Cells were incubated with LAMTOR5-AS1 sense probes. After DAPI staining, fluorescence was observed under a fluorescence microscope. Scale bar, 10 μm. NC FISH probe and 18S FISH probe were used as negative and cytoplasm control, respectively. RNA fluorescence in situ hybridization results showed that LAMTOR5-AS1 was localized in both the cytoplasm and nucleus, with cytoplasm localization being predominant. **B** The protein levels of KEAP1 and NRF2 determined by western blot in the three different regions of siRNAs of KEAP1 and the smart silencer of LAMTOR5-AS1 (ss-LAM) transfected into G-292 cells versus the negative control (NC). The levels of KEAP1 and NRF2 protein levels determined in SJSA-1 cells transfected with LAMTOR5-AS1-overexpressing lentivirus (LAM-OE) versus the negative control (NC-OE) analyzed by western blot. **C** The immunofluorescence of NRF2 and KEAP1 in the smart silencer of LAMTOR5-AS1 (ss-LAM) transfected into HEK293T cells versus the NC-transfected cells (ss-NC). **D** The NRF2 relative transcriptional activity was evaluated with the reporter construct pGL3 plasmid, ARE promoter-wt plasmid, ARE promoter-mut plasmid, NRF2-overexpressing plasmid or LAMTOR5-AS1 overexpression plasmid with the negative control (NC) were transfected into HEK293T cells. Both the firefly and Renilla luciferase activities were measured after 48 h transfection. n.s, no statistical significance; ***p-value < 0.001. **E** The NRF2 relative transcriptional activity was evaluated with the reporter construct pGL3 plasmid, ARE promoter-wt plasmid, ARE promoter-mut plasmid, si-KEAP1, NRF2-overexpressing plasmid, KEAP1 overexpression plasmid or LAMTOR5-AS1 overexpression plasmid with the negative control (NC) were transfected into HEK293T cells. Both the firefly and Renilla luciferase activities were measured after 48 h transfection. n.s, no statistical significance; *p-value < 0.05; **p-value < 0.01; ***p-value < 0.001. **F**, **G** Immunoprecipitation assays were used to analyze the effects of LAMTOR5-AS1 on the activity of KEAP1 and NRF2, the interaction between P62 and KEAP1, and the interaction between KEAP1 and NRF2. The HEK293T cells transfected with the smart silencer of LAMTOR5-AS1 (ss-LAM) versus the negative control (ss-NC), or transfected with LAMTOR5-AS1-overexpressing lentivirus (LAM-OE) versus the negative control (NC-OE), co-IP precipitant by KEAP1 (**F**). The HEK293T cells transfected with the smart silencer of LAMTOR5-AS1 (ss-LAM) versus the negative control (ss-NC), or transfected with LAMTOR5-AS1-overexpressing lentivirus (LAM-OE) versus the negative control (NC-OE), and cotransfected with HA-NRF2, Flag-KEAP1 or GFP-P62 overexpression plasmid, Co-IP precipitant by Flag-KEAP1 to confirm the effect of LAMTOR5-AS1 on the binding of NRF2 with KEAP1 (**G**).

Fluorescence in situ hybridization (FISH) assays showed that LAMTOR5-AS1 transcripts were observed in both the cytoplasm and nucleus, which means that LAMTOR5-AS1 may also regulate the transcriptional activity of NRF2 (Fig. 6A). As recent studies have shown that KEAP1 is associated with the ubiquitination degradation and transcriptional activity of NRF2 [27, 30], we further studied whether LAMTOR5-AS1 could affect its expression and transcriptional activity. Therefore, we analyzed the expression of KEAP1 and NRF2 in G-292 and SJSA-1 cells by western blotting or immunofluorescence analysis. The results showed that the expression levels of NRF2 were upregulated in response to LAMTOR5-AS1 overexpression but decreased following LAMTOR5-AS1 knockdown (Fig. 6B, C).

Moreover, to confirm whether LAMTOR5-AS1 could affect the transcriptional activity of NRF2, we constructed an ARE-promoter-luc plasmid and performed dual-luciferase reporter assays. Cotransfection of ARE-promoter-luc and pCDNA3.1-NRF2 promoted luc expression, while cotransfection of ARE-promoter-luc, pCDNA3.1-NRF2, pCDNA3.1 and pCDNA3.1-LAMTOR5-AS1 inhibited luc expression (Fig. 6D). These results suggest that LAMTOR5-AS1 inhibits the transcriptional activity of NRF2 by direct binding. Furthermore, the dual-luciferase reporter assays revealed that KEAP1 inhibited the transcriptional activity of NRF2 (Fig. 6E).

Moreover, we found that downregulation of LAMTOR5-AS1 impedes the binding of NRF2 and KEAP1, while overexpression of LAMTOR5-AS1 promotes their interactions. However, the interaction between P62 and KEAP1 was not affected by LAMTOR5-AS1 (Fig. 6F, G). These results demonstrate that LAMTOR5-AS1 inhibits NRF2 activity by enhancing the interaction between NRF2 and KEAP1. However, the binding of LAMTOR5-AS1 to NRF2 prevents NRF2 from KEAP1-induced ubiquitination.

### Downregulation of LAMTOR5-AS1 activates the NRF2/HO-1 pathway

**Fig. 7:**
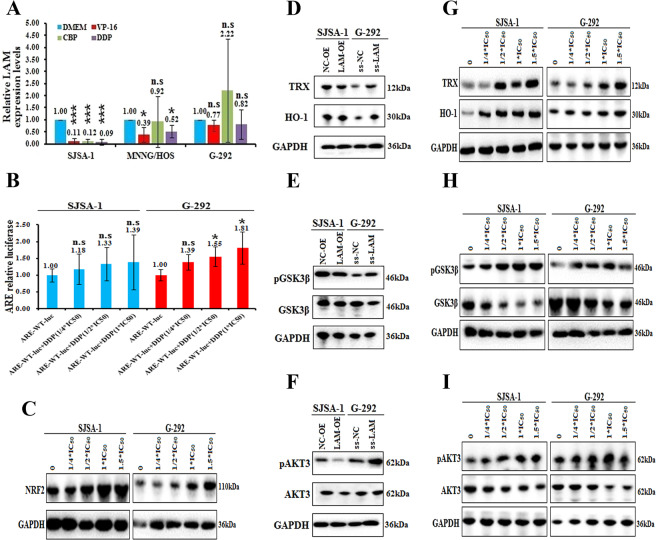
Downregulation of LAMTOR5-AS1 activates the NRF2/HO-1 pathway. **A** The levels of LAMTOR5-AS1 determined by real-time PCR in SJSA-1, MNNG/HOS and G-292 cells, which were treated with the IC_50_ dose of VP-16, CBP and DDP, respectively, with the DMEM as their control. n.s, no statistical significance; *p-value < 0.05; ***p-value < 0.001. **B** The ARE relative luciferase of SJSA-1 and G-292 cells transfected with pGL3-basic-ARE-wt after 24 h; two cell types were treated with the 1/4 x IC_50_, 1/2 x IC_50_ and 1 x C_50_ dose of DDP and with DMEM as the negative control. n.s, no statistical significance; *p-value < 0.05. **C** The protein expression levels of NRF2 in SJSA-1 and G-292 cells treated with the 1/4 x IC_50_, 1/2 x IC_50_, 1 x IC_50,_ and 1.5 x IC_50_ dose of DDP with DMEM as the negative control. (**D**)The protein expression levels of TRX and HO-1 in SJSA-1 cells transfected with LAMTOR5-AS1-overexpressing lentivirus (LAM-OE) versus the negative control (NC-OE), and transfected in G-292 cells with the smart silencer of LAMTOR5-AS1 (ss-LAM) versus the negative control (ss-NC). **E** The protein expression levels of phosphorylated GSK3β and GSK3β in SJSA-1 cells transfected with LAMTOR5-AS1-overexpressing lentivirus (LAM-OE) versus the negative control (NC-OE), and transfected in G-292 cells with the smart silencer of LAMTOR5-AS1 (ss-LAM) versus the negative control (ss-NC). **F** The protein expression levels of phosphorylated AKT3 and AKT3 in SJSA-1 cells transfected with LAMTOR5-AS1-overexpressing lentivirus (LAM-OE) versus the negative control (NC-OE), and transfected in G-292 cells with the smart silencer of LAMTOR5-AS1 (ss-LAM) versus the negative control (ss-NC). **G** The protein expression levels of TRX and HO-1 in SJSA-1 and G-292 cells, which were treated with the 1/4 x IC_50_, 1/2 x IC_50_, 1 x IC_50_, and 1.5 x IC_50_ dose of DDP and with DMEM as the negative control. **H** The protein expression levels of phosphorylated GSK3β and GSK3β in SJSA-1 and G-292 cells, which were treated with the 1/4 x IC_50_, 1/2 x IC_50_,1 x IC_50_, and 1.5 x IC_50_ dose of DDP and with DMEM as the negative control. **I** The protein expression levels of phosphorylated AKT and AKT3 in SJSA-1 and G-292 cells, which were treated with the 1/4 x IC_50_, 1/2 x IC_50_,1 x IC_50,_ and 1.5 x IC_50_ dose of DDP and with DMEM as the negative control.

We cultured SJSA-1, G-292 and MNNG/HOS cells with VP-16, CBP or DDP and found that the expression of LAMTOR5-AS1 was downregulated in these three cell lines treated with VP-16 or DDP (Fig. 7A). Moreover, we found that DDP-induced LAMTOR5-AS1 downregulation could promote NRF2 expression and activation in a concentration-dependent manner by dual-luciferase reporter gene detection and western blotting (Fig. 7B, C). In addition, downregulation of LAMTOR5-AS1 significantly promoted the expression of HO-1 and thioredoxin reductases-1 (TRX), which are downstream genes of NRF2 [27, 31], whereas upregulation of LAMTOR5-AS1 had the opposite effect (Fig. 7D).

Glycogen synthase kinase-3 beta (GSK3β) and AKT3 are considered to be the upstream molecules of NRF2 [27], and dephosphorylation of GSK3β could promote the phosphorylation of NRF2 and lead to ubiquitination degradation, while activation of the PI3K/AKT pathway inhibits GSK3β function by promoting its phosphorylation. To further identify the role of LAMTOR5-AS1 in NRF2 upstream regulatory molecules, we focused on the expression and phosphorylation of GSK3β and AKT3 induced by LAMTOR5-AS1. The results showed that LAMTOR5-AS1 knockdown promoted the phosphorylation of AKT3 and GSK3β, while LAMTOR5-AS1 overexpression inhibited the phosphorylation of GSK3β and AKT3 (Fig. 7E, F). Furthermore, downregulation of LAMTOR5-AS1 inhibits the expression of GSK3β (Fig. 7E).

Western blot analysis further showed that the expression levels of TRX, HO-1, phosphorylated AKT3 and phosphorylated GSK3β increased significantly in a DDP concentration-dependent manner. However, the expression of GSK3β decreased significantly under DDP induction (Fig. 7G-I). These data support the role of LAMTOR5-AS1 as a drug-sensitive molecule that regulates the expression and activity of NRF2 and its upstream and downstream molecules.

### LAMTOR5-AS1 inhibits OS drug resistance in vivo

**Fig. 8:**
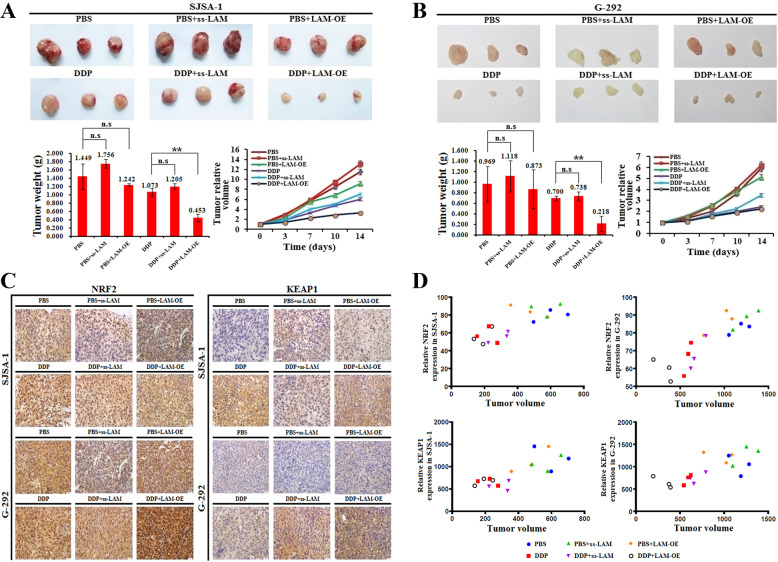
LAMTOR5-AS1 inhibits OS drug resistance in vivo. **A** The effect of LAMTOR5-AS1 and DDP on tumor formation, tumor weight, and tumor volume change in a nude mouse SJSA-1-derived xenograft model. Representative images of tumors from PBS, PBS with the smart silencer of LAMTOR5-AS1 (PBS + ss-LAM) and PBS with the LAMTOR5-AS1-overexpressing lentivirus (PBS + LAM-OE); the corresponding three groups were the DDP, DDP with silencer of LAMTOR5-AS1 (DDP + ss-LAM), DDP with LAMTOR5-AS1-overexpressing lentivirus (DDP + LAM-OE), respectively (*n* = 3 for each group). **B** The effect of LAMTOR5-AS1 and DDP on tumor formation, tumor weight and tumor volume changes in a nude mouse G-292-derived xenograft model. Representative images of tumors from PBS, PBS with the smart silencer of LAMTOR5-AS1 (PBS + ss-LAM) and PBS with the LAMTOR5-AS1-overexpressing lentivirus (PBS + LAM-OE); the corresponding three groups were DDP, DDP with silencer of LAMTOR5-AS1 (DDP + ss-LAM), DDP with LAMTOR5-AS1 overexpression lentivirus (DDP + LAM-OE), respectively (*n* = 3 for each group). **C** The protein levels of NRF2 and KEAP1 were determined by IHC (magnification: ×200) in the six groups: PBS, PBS with the smart silencer of LAMTOR5-AS1 (PBS + ss-LAM), PBS with the LAMTOR5-AS1-overexpressing lentivirus (PBS + LAM-OE), DDP, DDP with silencer of LAMTOR5-AS1 (DDP + ss-LAM), and DDP with LAMTOR5-AS1-overexpressing lentivirus (DDP + LAM-OE). **D** The correlation analysis between tumor growth and expression of NRF2 and KEAP1 in SJSA-1 and G-292 cells, which were treated with PBS, PBS with the smart silencer of LAMTOR5-AS1 (PBS + ss-LAM), PBS with the LAMTOR5-AS1-overexpressing lentivirus (PBS + LAM-OE), DDP, DDP with silencer of LAMTOR5-AS1 (DDP + ss-LAM), and DDP with LAMTOR5-AS1-overexpressing lentivirus (DDP + LAM-OE).

We then constructed a tumor xenograft mouse model to test the role of LAMTOR5-AS1 on drug resistance in vivo. The results suggest that LAMTOR5-AS1 overexpression promoted the effects of DDP on the inhibition of tumor formation, tumor weight and tumor volume, while LAMTOR5-AS1 knockdown promoted OS cell resistance against DDP (Fig. 8A, B).

Next, we detected the expression of NRF2, KEAP1, HO-1, AKT, pAKT, GSK3β, pGSK3β, TRX and P62 by immunohistochemistry. The results showed that the expression of NRF2 was the highest in the DDP + LAMTOR5-AS1 overexpression (DDP + LAM-OE) group, while the expression of NRF2 in the DDP group was higher than that in the DDP + smart silencer of LAMTOR5-AS1 (DDP + LAM-OE) group, and the PBS + ss-LAM group showed the lowest expression in a nude mouse G-292-derived or SJSA-1 xenograft model (Fig. 8C, left). Notably, under the same treatment conditions, KEAP1 expression was different in nude mouse G-292- and SJSA-1-derived xenograft models. The expression level of KEAP1 was highest in the DDP + LAM-OE group in a nude mouse G-292- or SJSA-1-derived xenograft model, but the expression of KEAP1 in the DDP group was lower than that in the DDP + ss-LAM group in a nude mouse G-292-derived xenograft model and was higher than that in a nude mouse SJSA-1-derived xenograft model (Fig. 8C, right). In G-292 cells, although downregulation of LAMTOR5-AS1 expression could promote NRF2 activity, upregulation of KEAP1 expression induced by DDP could promote the ubiquitination of NRF2 and inhibit its transcriptional activity. However, in SJSA-1 cells, the upregulation of KEAP1 expression caused by LAMTOR5-AS1 deletion was not significant. According to the above results, we speculated that differentially expressed KEAP1 might lead to a difference in drug resistance between G-292 and SJSA-1 cells. To further clarify the relationship between tumor size and the expression of NRF2 or KEAP1, we constructed the relationship between gene expression and tumor size (Fig. 8D).

## Discussion

LncRNAs can regulate gene expression via various aspects, including chromatin modification and transcriptional and posttranscriptional processing [32]. Accumulating studies have revealed the extensive regulatory functions of lncRNAs in tumorigenesis and progression [33]. Long-noncoding RNA LAMTOR5 antisense RNA 1 (LAMTOR5-AS1) has been certified as a risk predictor and diagnostic biomarker of prostate cancer and non-small cell lung cancer [34]. However, the expression and exact roles of LAMTOR5-AS1 in osteosarcoma remain unclear. In this study, we identified a series of differentially expressed lncRNAs in OS cells. Among these lncRNAs, we focused on LAMTOR5-AS1, which is upregulated in G-292 cells compared to that in SJSA-1 cells, and both downregulated by DPP. LAMTOR5-AS1 is named after LAMTOR5 antisense RNA 1 [35], which comes from the reverse transcription of the LAMTOR5 parent gene. However, the detailed role and function of LAMTOR5-AS1 in OS remain to be clarified.

We showed that LAMTOR5-AS1 in OS tumor tissues was significantly downregulated compared to that in adjacent normal muscle tissue by analyzing TCGA and GEO database data. Functional experiments showed that LAMTOR5-AS1 inhibited OS tumorigenesis and metastasis in vitro and in vivo. We also observed that LAMTOR5-AS1 could promote the chemosensitivity of OS cells by promoting OS cell apoptosis and inhibiting proliferation. However, no significant correlation was found between the expression of LAMTOR5-AS1 and the prognosis of patients with OS. We analyzed whether the expression of LAMTOR5-AS1 was related to the prognosis of patients with other types of tumors by analyzing TCGA database and found that OS patients with higher expression of LAMTOR5-AS1 had a better prognosis. We thus speculate that LAMTOR5-AS1 has different effects, which depend on the heterogeneity of different tumors or even the same tumors in different individuals. OS is a high incidence disease in adolescents. When we limited the age of OS patients to 10–20 years old [36], we found that patients with a high expression of LAMTOR5-AS1 had a better prognosis than patients with a lower expression of LAMTOR5-AS1. Therefore, we speculate that the individual differences of different age groups of OS patients may be an important reason for the low correlation between LAMTOR5-AS1 expression and prognosis.

NRF2 is an important transcription factor involved in regulating the cellular oxidative stress response, and it is also a central regulator for maintaining intracellular redox homeostasis [23, 37, 38]. NRF2 can reduce the cell damage caused by ROS and electrophilicity by regulating the constitutive and inducible expression of a series of antioxidant proteins, keeping the cells in a stable state and maintaining the dynamic redox balance [27, 39]. Our results reveal a new mechanism of LAMTOR5-AS1 in DDP resistance of osteosarcoma cells. The expression of LAMTOR5-AS1 in osteosarcoma was significantly lower than that in normal surrounding tissues. More interestingly, DDP can induce further reduction of LAMTOR5-AS1 expression in OS cells. The above results reveal an interesting phenomenon that LAMTOR5-AS1 may play an inhibitory role in osteosarcoma and drug tolerance. The cytological experiment of overexpression or knock down LAMTOR5-AS1 in OS cells also confirmed the function of LAMTOR5-AS1. What’s more, we found that LAMTOR5-AS1 can promote NRF2 protein expression by inhibiting its ubiquitination. This phenomenon makes us very confused, because NRF2 is the key factor of chemoresistance of tumor cells, and the role of LAMTOR5-AS1 seems to help NRF2 protein maintain stability, which is contrary to the inhibition of drug resistance of OS cells by LAMTOR5-AS1. So, we speculate that there may be two aspects of the functions of LAMTOR5-AS1 on NRF2. Not surprisingly, further studies found that the activity detection experiment of transcription factors revealed that LAMTOR5-AS1 overexpression could inhibit NRF2 activity. However, how LAMTOR5-AS1 inhibits both ubiquitination and transcriptional activity of NRF2 remains unclear. The localization of lncRNA in cells is closely related to the regulation of lncRNA. LncRNA often plays a regulatory role as a miRNA sponger or protein regulator in the cytoplasm, while lncRNA plays a transcriptional regulatory role in the nucleus. Interestingly, through fluorescence in situ hybridization and nucleocytoplasmic separation experiments, we revealed that LAMTOR5-AS1 was distributed in both cytoplasm and nucleus, and the distribution in cytoplasm was higher than that in nucleus. Our study found that there was mutual binding between LAMTOR5-AS1 and NRF2 protein. Through this direct binding mechanism, we speculate that LAMTOR5-AS1 may change the interaction environment between NRF2 protein and other proteins, and Co-IP experiment showed that LAMTOR5-AS1 could promote the interaction between NRF2 and KEAP1without affecting the binding between KEAP1 and P62. This gives us a hypothesis that the inhibition of NRF2 function by LAMTOR5-AS1 seems to depend on promoting the interaction between KEAP11 and NRF2. KEAP1 was found to regulate the ubiquitination degradation and transcriptional activity of NRF2 [40]. It forms a homodimer and combines with NRF2 to promote its ubiquitination, while phosphorylated P62 binds to KEAP1 and inhibits its interaction with the N-terminus of NRF2, thus removing the ubiquitination of NRF2 [27, 39, 41]. However, there is still a phenomenon that cannot be clearly explained, knocking down KEAP1 expression still cannot effectively improve the inhibition of NRF2 activity by LAMTOR5-AS1, which needs further research. What’s more, this phenomenon shows higher efficiency for the regulation of NRF2 protein by LAMTOR5-AS1. For the biological activities of cells, the time and material cost of de novo synthesis of some important proteins may be much higher than that of functional inhibition. When cells encounter adverse environments such as heat shock and chemotherapy drugs, these proteins can be quickly produced and help cells resist the adverse environment [42, 43]. Tumor cells may promote an increase in drug resistance by utilizing and strengthening this ability.

**Fig. 9:**
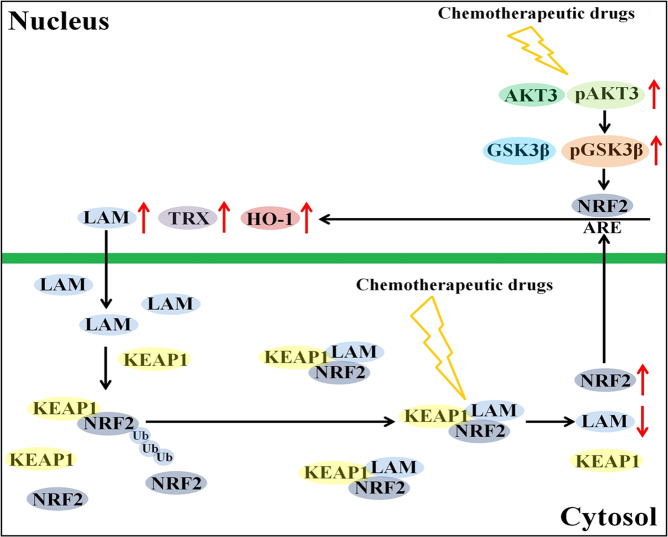
Schematic illustration of proposed model depicting the roles of LAMTOR5-AS1 in the chemotherapy. On the one hand, LAMTOR5-AS1 acts as a bridge between KEAP1 and NRF2 and promotes their interaction. On the other hand, LAMTOR5-AS1 inhibits the ubiquitination of NRF2 by binding to NRF2. When cells treated with chemotherapeutic drugs, the redox balance of tumor cell changed, LAMTOR5-AS1 was degraded and NRF2 was released. The released NRF2 entered into nucleus and regulated the expression of related genes. With the enhancement of NRF2 activity and the resolution of crisis, NRF2 feedback regulated LAMTOR5-AS1, the overexpressed LAMTOR5-AS1 transformed into cytoplasm and inhibited the activity of NRF2 to regain the reactive oxygen species balance.

To further study the effect of LAMTOR5-AS1 on NRF2 transcriptional activation, we investigated the expression changes in the NRF2 downstream genes HO-1 and TRX and identified the effect of NRF2 on downstream gene activation under drug or LAMTOR5-AS1 downregulation. The increase in oxidative damage induced by chemotherapeutic drugs participates in killing tumor cells, while upregulation of HO-1 and TRX can effectively resist the oxidative damage faced by cells and inhibit the cytotoxic effect of chemotherapeutic drugs on tumor cells [44–46]. We also studied the changes in the NRF2 upstream genes GSK3β and AKT3 and observed a phenomenon [27, 47, 48]. Compared to that in G-292 cells, the expression of nonphosphorylated GSK3β was significantly decreased in SJSA-1 cells induced by DDP or downregulation of LAMTOR5-AS1. GSK3β also plays an important role in the regulation of NRF2 ubiquitination. Nonphosphorylated GSK3β can promote NRF2 phosphorylation, while phosphorylated NRF2 can recruit the b-trcp-cul1 protein complex to promote its own ubiquitination degradation. However, we found that upregulation of LAMTOR5-AS1 did not alter GSK3β expression, while downregulation of LAMTOR5-AS1 resulted in a decrease in nonphosphorylated GSK3β. This phenomenon is inconsistent with the hypothesis that LAMTOR5-AS1 overexpression inhibits NRF2 ubiquitination, while LAMTOR5-AS1 downregulation promotes NRF2 ubiquitination. We hypothesized that LAMTOR5-AS1 inhibited NRF2 ubiquitination mainly in the NRF2/KEAP1 pathway, while cell resistance to DDP has two pathways. On the one hand, DDP-induced downregulation of LAMTOR5-AS1 can promote NRF2 activity; on the other hand, DDP can inhibit the ubiquitination of NRF2 by inhibiting the expression of nonphosphorylated GSK3β (Fig. 9).

Taken together, Transcription factors mainly mediate the transcriptional expression of downstream genes in the nucleus. The post-modification of transcription factors often changes their localization in the nucleus. In the activation process of TGFβ-mediated SMAD4/SMAD2/3 pathway, phosphorylation of SMAD2/3 is required for the interaction with SMAD4, and thus significantly affects the formation and nucleation of SMAD4/SMAD2/3 protein complex [49].

Notably, the current study also found that NRF2 entry into the nucleus is regulated by KEAP1. KEAP1 binding to NRF2 will prevent NRF2 from entering into the nucleus, which in return affects the exertion of NRF2 activity [50]. Our study found that LAMTOR5-AS1 can bind to NRF2 in the cytoplasm, inhibit the degradation of NRF2 and thus maintain the stability of NRF2 protein, prevent NRF2 from entering into the nucleus. Generally, LAMTOR5-AS1 has a significant inhibitory effect on the activity of NRF2.

Via the immunofluorescence and nucleocytoplasmic separation experiments, we found that the nuclear localization of NRF2 was significantly reduced under the action of LAMTOR5-AS1. It was speculated that LAMTOR5-AS1 inhibits the function of NRF2 via impeding the nuclear localization of NRF2, but not its expression level.

Moreover, we found that NRF2 has a feedback regulation effect on LAMTOR5-AS1, which has a profound effect on the redox homeostasis control. The chemotherapeutic drugs will induce the cells into a state of oxidative stress. At this time, the LAMTOR5-AS1/NRF2/KEAP1 complex disintegrates and releases NRF2 into the cytoplasm. With the increase of NRF2 concentration, NRF2 enters the nucleus, LAMTOR5-AS1 binds to the promoter of NRF2 in the nucleus, promotes the NRF2 activity, which regulates the expression of related genes to relieve the oxidative stress. When the oxidative stress is relieved, NRF2 feedback up-regulates the expression of LAMTOR5-AS1, which enters into the cytoplasm and reforms the complex LAMTOR5-AS1/NRF2/KEAP1 to achieve redox homeostasis.

## Materials and methods

### Cell lines and culture conditions

Human OS cell lines (SJSA-1, MNNG/HOS and G-292) were purchased from American Type Culture Collection (ATCC), and a human embryonic kidney cell line (HEK293T) was purchased from the Institute of Biochemistry and Cell Biology of the Chinese Academy of Sciences (SIBCB). Cells were cultured in DMEM or RPMI 1640 (BI, China) medium supplemented with 10% fetal bovine serum (PAN, China), 100 U/ml penicillin, and 100 mg/ml streptomycin (WISENT, China) in humidified air at 37 °C with 5% CO_2_. All cell lines tested negative for mycoplasma contamination.

### RNA sequencing

Total RNA of SJSA-1 or G-292 cells was extracted by TRIzol reagent, and cDNA was produced by gene-specific primers or random primers. The Illumina-HiSeq 4000 system was used for RNA sequencing, and the Illumina-HiSeq 2000 system was used for library sequencing (BGI, China). Briefly, rRNA was removed prior to strand-specific library construction. Quality control assessment and quantification of sample libraries were performed using an Agilent 2100 Bioanalyzer and ABI StepOnePlus Real-Time PCR System before library sequencing using an Illumina-HiSeq^TM^ 2000 in 100 bp single read mode. Gene expression levels were measured by the number of uniquely mapped fragments per kilobase of transcript per million mapped reads (FPKM), and then lncRNA-seq was performed to screen the differentially expressed lncRNAs in chemoresistant SJSA-1 cells versus chemosensitive G-292 cells [26] (SI Appendix, Table S1) (GEO accession number: GSE153786).

### Plasmid constructs

The LAMTOR5-AS1 promoter sequence was synthesized by General Biotechnology (China), digested and ligated to a PGL3-basic vector, and the NRF2 fragment was digested and ligated to the p3xFLAG-*Myc*-CMV vector for luciferase assays. The NRF2 fragment was digested and ligated to the pCMV-HA vector, the KEAP1 fragment was digested and ligated to the p3xFLAG-*Myc*-CMV vector, and the P62 fragment was digested and ligated to the pEGFP-C1 vector for co-IP assays. The LAMTOR5-AS1, KEAP1 and NRF2 sequences were amplified by PCR and cloned into the eukaryotic expression vector pcDNA3.1 and the lentiviral expression vector pHBLV-CMV-MCS-3xflag-EF1-mCherry-t2a-puro. Detailed information regarding the primers used for plasmid constructs is depicted in SI Appendix, Table S2.

### Biotin RPD assays

RPD assays were performed using the Pierce Magnetic RNA-Protein Pull-Down Kit (Thermo Fisher) according to the manufacturer’s instructions. Briefly, the LAMTOR5-AS1 sequence was transcribed in vitro with a biotin RNA-labeling mix according to the manufacturer’s instructions. Twenty microliters of streptavidin-conjugated magnetic beads was washed with RPD buffer, 3 μg of LAMTOR5-AS1 RNA sense probe (1 μg for probes 1, 2, and 3) and 3 μg of LAMTOR5-AS1 RNA antisense probe (1 μg for probes 1, 2, and 3) were added, and the cells were incubated at 4 °C for 1–3 h and the total cell lysates were incubated at room temperature for 2 h. The bead-RNA-protein complexes were washed with 1 × binding-washing buffer four times. The proteins were precipitated and diluted in protein lysis buffer. Finally, the retrieved proteins were measured by real-time PCR and/or western blot analysis. Detailed information regarding the primers used for real-time PCR analysis is depicted in SI Appendix, Table S2.

### Mass spectrometry

The eluted proteins from LAMTOR5-AS1 RPD assays were identified using a gel-based liquid chromatography-tandem mass spectrometry approach [51]. A Mascot database search was used to visualize and validate the results.

### RIP assays

RIP was performed with RIP Kit (Millipore, America) according to the instructions provided by the manufacturer [52]. Briefly, ~2–4 × 10^7^ G-292 cells were lysed in hypotonic buffer supplemented with RNase inhibitor and protease inhibitor before centrifugation. The lysates were incubated with magnetic beads and then coated with the indicated antibodies for 4 h or overnight at 4 °C. After extensive washing using RIP wash buffer, the bead-bound immunocomplexes were treated with proteinase K for 30 min at 55 °C. Samples were centrifuged and placed on a magnetic separator, and supernatants were used to extract RNA by Kit (Bioline, Italy). Purified RNAs were then subjected to PCR analysis. Detailed information regarding the primers used for PCR analysis is depicted in SI Appendix, Table S2.

### ChIP assays

ChIP assays were performed using a Millipore ChIP Kit (Millipore, America) according to the manufacturer’s instructions [52]. Bound DNA fragments were subjected to qPCR using the specific primers shown in SI Appendix, Table S1.

### Luciferase reporter assays

NRF2 transcriptional activity was evaluated with the reporter construct pGL3 (Invitrogen, America), which contains two copies of antioxidant response elements (AREs) that drive the expression of the luciferase reporter luc gene. The NRF2 target sequence of the ARE promoter was cloned into the 3’ end of the luciferase-coding sequence of pGL3 to construct ARE promoter-wt, and the NRF2 target sequence was deleted to construct ARE promoter-mut. The constructs were confirmed by DNA sequencing, and HEK293T cells were seeded in 96-well plates at ~1 × 10^4^ cells per well and transfected with a mixture of ARE promoter-wt or mut, LAMTOR5-AS1, NRF2 or KEAP1 overexpression plasmid, si-KEAP1, Renilla and the negative control with the Lipofectamine 2000 transfection kit according to the manufacturer’s instructions. Both firefly and Renilla luciferase activities were measured 24 h after transfection using the Dual-Luciferase Reporter Assay System (Promega, America) and a Promega GloMax 20/20 luminometer. The relative firefly luciferase activities of the UTR construct and pathway reporter constructs were analyzed as previously reported [53]. Detailed information regarding the primers used for plasmid construction is depicted in SI Appendix, Table S2.

### FISH assays

The cells were washed with PBS and fixed with 4% paraformaldehyde for 15 min at room temperature. Cells were permeabilized into PBS containing 0.5% Triton-X-100 for 15 min at 4 °C and then washed in PBS for 5 min. After that, 70%, 85% and 100% ethanol were used for dehydration for 3 min. The FISH probe was hybridized in a humid chamber at 75 °C for 5 min for denaturation, and then the fluorescence in situ hybridization kit (GenePharma, China) was used overnight in the dark at 37 °C. The slides were washed three times with buffer F (20× SSC with 0.1% Tween-20). The slides were washed at room temperature for 5 min with 2 × washing buffer C (40× SSC) and washing buffer C (20× SSC). The slides were stained with DAPI for 20 min in the dark. The LAMTOR5-AS1, NRF2 and KEAP1 FISH probes were designed and synthesized with Genemarma (China). NC FISH probe and 18S FISH probe were used as negative and cytoplasmic controls, respectively. All images were obtained by fluorescence microscopy or confocal microscopy (Nikon, Japan). Detailed information regarding the probes is depicted in SI Appendix, Table S2, and detailed information regarding the localization of NRF2 and KEAP1 is depicted in SI Appendix, Fig. S2.

### Co-IP assays

HEK293T cells stably transfected with 5 μg LAMTOR5-AS1 overexpression plasmid or 20 μl si-LAMTOR5-AS1 (20 μM) with 5 μg HA-NRF2, Flag-KEAP1, or GFP-P62 overexpression plasmid were resuspended in immunoprecipitation buffer (20 mM Tris-HCl, 150 mM NaCl, 2.5 mM MgCl_2_, 1 mM EDTA, 10% glycerol, 0.5% NP-40, 0.5% Triton-X-100, pH = 7.5). Co-IP precipitates assayed with the anti-KEAP1, anti-Flag-KEAP1 or IgG antibody was immunoblotted with an anti-NRF2, anti-KEAP1, anti-P62 or anti-Flag antibody to confirm the effect of LAMTOR5-AS1 on the binding of NRF2 to KEAP1. Detailed information regarding the primers used for plasmid construction is depicted in SI Appendix, Table S2, and the detailed antibody information is depicted in SI Appendix, Tables S3 and S4.

### Western blot assays

Cells were harvested and washed twice with cold PBS. After adding lysis buffer (60 mM Tris-HCl, pH 6.8, 2% SDS, 20% glycerol, 0.25% bromophenol blue and 1.25% 2-mercaptoethanol), samples were lysed and heated for 10 min at 95 °C, followed by centrifugation at 4 °C and 13,000 rpm for 30 min. Whole-cell proteins were subjected to sodium dodecyl sulfate-polyacrylamide gel electrophoresis (SDS-PAGE) and subsequently transferred to nitrocellulose membranes. Membranes were blocked with 5% nonfat milk at room temperature for 1 h or at 4 °C overnight and then incubated with the appropriate antibody. Images were captured by the Image Reader. Detailed information regarding the antibody is depicted in SI Appendix, Table S3 and S5. Detailed information regarding the full-length gels is depicted in SI Appendix, Figs. S3–S6.

### Immunofluorescence assays

For immunofluorescence analysis, ss-LAMTOR5-AS1-transfected cells were fixed with 4% paraformaldehyde at room temperature for 20 min. Then, the cells were washed three times with PBS, permeabilized with 0.1% Triton-X-100 for 20 min and blocked in 5% bovine serum albumin (BSA) at room temperature for 20 min. Primary antibodies against NRF2 or KEAP1 were added to coverslips at 4 °C overnight, and fluorescently-labeled secondary antibodies were added to coverslips for 1 h at room temperature. After 10 min of treatment with DAPI, the images were observed with a Car Zeiss microscope. Detailed information regarding the antibodies is depicted in SI Appendix, Table S3.

### Immunohistochemistry assays

The protein expression levels of NRF2, KEAP1, P62, phosphorylated AKT3 (pAKT3), AKT3, phosphorylated GSK3β (pGSK3β), GSK3β, TRX and HO-1 were determined by immunohistochemistry (IHC). IHC staining was performed on 4-mm sections of paraffin-embedded tissue samples. Briefly, the slides were incubated with the appropriate antibody at 4 °C overnight. The subsequent steps were performed using the GTVision III Detection System/Mo&Rb (GeneTech, China). Detailed information regarding the antibody is depicted in SI Appendix, Table S3.

### Tumor xenograft mouse model

Four-week-old male BALB/c nude mice were purchased from Zhejiang Weitong Lihua Laboratory Animal Technology Co., Ltd. The nude mice were kept in the SPF Animal Laboratory of China University of Science and Technology. Animal experiments were conducted in accordance with the National Guidelines for the Health Use of Laboratory Animals. Animal research was approved by the Biomedical Ethics Committee of China University of Science and Technology. The procedures for mouse experiments were implemented in accordance with the Regulations on the Administration of Laboratory Animals approved by the State Council.

SJSA-1 or G-292 cells expressing ss-LAMTOR5-AS1 or LAMTOR5-AS1-OE or the control were embedded in BD Matrigel Matrix and subcutaneously injected into the anterior dorsal flanks of each mouse, each group was repeated three times. After tumors formed, the tumor size was measured every 2 days with calipers. In drug therapy experiments, mice were divided into two groups: the administration group intraperitoneally receiving DDP (75 μg/mouse), and the control group treated with PBS. These injections were performed four times at an interval of 2 days between each injection (i.e., days 7, 10, 13, etc.). The mice were humanely sacrificed after four drug injections, ~25 days later, and their subcutaneous tumors were isolated, weighed and imaged. The tumor weight was described as the mean ± S.D., and the tumor volume was calculated by the equation *V* = 0.5 × *D* × *d*^2^ (*V*: volume, *D*: longitudinal diameter, *d*: latitudinal diameter).

The protein expression of NRF2 and KEAP1 in the xenograft tumors of nude mice immobilized with formalin and paraffin was determined by immunochemical methods. In a 750 W microwave oven, the dewaxed sections were pretreated in citrate buffer (pH 6) for 5 min for antigen retrieval and treated with a hypersensitive link marker detection system (Biogenex, America). Next, 3-amino-9-ethylcarbazole was used as the developing carrier. After the slides were stained with Invitrogen reagents, they were loaded into the water-borne installation medium. Photographs were taken with a Leica DM 4000b microscope, the relative levels of each protein were calculated with Leica software, and the percentage of simulated treatment tumors relative to chemotherapy treatment tumors was calculated and plotted. Detailed information regarding the expression of the P62, AKT3, pAKT3, GSK3β, pGSK3β, TRX and HO-1 proteins is depicted in SI Appendix, Fig. S7.

### Statistical analysis

Statistical analysis was carried out using Microsoft Excel software and GraphPad Prism to assess differences between experimental groups. Statistical significance was analyzed by a two-tailed Student’s *t*-test. *p*-values < 0.05 were considered to be statistically significant: *p-value < 0.05; **p-value < 0.01; ***p-value < 0.001.

## Supplementary information

Authors email confirming changes

Supplementary materials
