# Supplementary material for: LAMTOR5-AS1 regulates chemotherapy-induced oxidative stress by controlling the expression level and transcriptional activity of NRF2 in osteosarcoma cells
Source: Cell Death Dis. 2021 Dec 3;12(12):1125. doi: 10.1038/s41419-021-04413-0 (PMC8642434; doi:10.1038/s41419-021-04413-0)
Supplement: Supplementary file 1 — Authors email confirming changes [file 41419_2021_4413_MOESM1_ESM.pdf]

Authors email confirming changes

1.Youguang Pu

Re: 请确认自己的姓名、邮箱、地址及排序（投稿的Cell Death & Disease文章已初步接收） 发起会议 精简信息

发件人： pyg

时　间： 2021年11月09日 09:53:19 (星期二)

收件人： Dabing H... <hdabing@ustc.edu.cn>

I have carefully proofread and confirmed that it is correct.

-----原始邮件-----
发件人:"Dabing Huang" <hdabing@ustc.edu.cn>
发送时间:2021-11-09 09:45:43 (星期二)
收件人: pyg <pyg@ustc.edu.cn>
抄送:
主题: 请确认自己的姓名、邮箱、地址及排序（投稿的Cell Death & Disease文章已初步接收）

各位参与人，我们投稿的Cell Death & Disease文章已初步接收。应期刊要求所有参与人需确认自己的姓名、邮箱、地址及排序，为尽快在线发表，请大家及时回复（我已认真校对，确认无误），我们收集到之后统一整理。谢谢

Dear participants, the cell death & disease articles submitted by us have been preliminarily accepted. As required by the journal, all participants need to confirm their name, email address and ranking. In order to publish online as soon as possible, please reply in time (I have carefully proofread and confirmed that it is correct), and we will sort it out after collecting it. thank you!

I have carefully proofread and confirmed that it is correct.

--
Department of Oncology, the First Affiliated Hospital of USTC, Division of Life Sciences and Medicine, University of Science and Technology of China, Hefei, Anhui Province, 230001, P.R.China.

2.Yiao Tan

Re:请确认自己的姓名、邮箱、地址及排序（投稿的Cell Death & Disease文章已初步接收） 发起会议 精简信息

发件人： 谈宜傲 <tanyiaoa@163.com>

时　间： 2021年11月09日 10:04:48 (星期二)

收件人： Dabing H... <hdabing@ustc.edu.cn>

I have carefully proofread and confirmed that it is correct.thank you!

在 2021-11-09 09:46:15，"Dabing Huang" <hdabing@ustc.edu.cn> 写道:

各位参与人，我们投稿的Cell Death & Disease文章已初步接收。应期刊要求所有参与人需确认自己的姓名、邮箱、地址及排序，为尽快在线发表，请大家及时回复（我已认真校对，确认无误），我们收集到之后统一整理。谢谢

Dear participants, the cell death & disease articles submitted by us have been preliminarily accepted. As required by the journal, all participants need to confirm their name, email address and ranking. In order to publish online as soon as possible, please reply in time (I have carefully proofread and confirmed that it is correct), and we will sort it out after collecting it. thank you!

I have carefully proofread and confirmed that it is correct.

--
Department of Oncology, the First Affiliated Hospital of USTC, Division of Life Sciences and Medicine, University of Science and Technology of China, Hefei, Anhui Province, 230001, P.R.China.

3.Chunbao Zang

回复：请确认自己的姓名、邮箱、地址及排序（投稿的Cell Death & Disease文章已初步接收） 发起会议 精简信息

发件人： zangchun... <zangchun...@ustc.edu.cn>

时 间： 2021年11月09日 10:00:02 (星期二)

收件人： hdabing <hdabing@ustc.edu.cn>

I have carefully proofread and confirmed that it is correct.

zangchunbao

邮箱：zangchunbao@ustc.edu.cn

发自 网易邮箱大师

---- 回复的原邮件 ----

发件人 Dabing Huang<hdabing@ustc.edu.cn>

日期 2021年11月09日 09:47

收件人 zangchunbao@ustc.edu.cn<zangchunbao@ustc.edu.cn>

主题 请确认自己的姓名、邮箱、地址及排序（投稿的Cell Death & Disease文章已初步接收）

各位参与人，我们投稿的Cell Death & Disease文章已初步接收。应期刊要求所有参与人需确认自己的姓名、邮箱、地址及排序，为尽快在线发表，请大家及时回复（我已认真校对，确认无误），我们收集到之后统一整理。谢谢

Dear participants, the cell death & disease articles submitted by us have been preliminarily accepted. As required by the journal, all participants need to confirm their name, email address and ranking. In order to publish online as soon as possible, please reply in time (I have carefully proofread and confirmed that it is correct), and we will sort it out after collecting it. thank you!

4.Fangfang Zhao

Re: 请确认自己的姓名、邮箱、地址及排序（投稿的Cell Death & Disease文章已初步接收） 发起会议 精简信息

发件人： zhaofangfang

时 间： 2021年11月09日 12:03:37 (星期二)

收件人： Dabing H... <hdabing@ustc.edu.cn>

I have carefully proofread and confirmed that it is correct, thank you!

在2021-11-09 09:47:46，Dabing Huanghdabing@ustc.edu.cn写道：

各位参与人，我们投稿的Cell Death & Disease文章已初步接收。应期刊要求所有参与人需确认自己的姓名、邮箱、地址及排序，为尽快在线发表，请大家及时回复（我已认真校对，确认无误），我们收集到之后统一整理。谢谢

Dear participants, the cell death & disease articles submitted by us have been preliminarily accepted. As required by the journal, all participants need to confirm their name, email address and ranking. In order to publish online as soon as possible, please reply in time (I have carefully proofread and confirmed that it is correct), and we will sort it out after collecting it. thank you!

I have carefully proofread and confirmed that it is correct.

--

Department of Oncology, the First Affiliated Hospital of USTC, Division of Life Sciences and Medicine, University of Science and Technology of China, Hefei, Anhui Province, 230001, P.R.China.

5.Cifeng Cai

回复：请确认自己的姓名、邮箱、地址及排序（投稿的Cell Death & Disease文章已初步接收） 发起会议 精简信息

发件人： 前世今生 <148256249@qq.com>

时 间： 2021年11月09日 11:41:48 (星期二)

收件人： Dabing H... <hdabing@ustc.edu.cn>

I have carefully proofread and confirmed that it is correct, thank you!

------------------ 原始邮件 ------------------

发件人: "Dabing Huang" <hdabing@ustc.edu.cn>;

发送时间: 2021年11月9日(星期二) 中午11:25

收件人: "前世今生"<148256249@qq.com>;

主题: 请确认自己的姓名、邮箱、地址及排序（投稿的Cell Death & Disease文章已初步接收）

各位参与人，我们投稿的Cell Death & Disease文章已初步接收。应期刊要求所有参与人需确认自己的姓名、邮箱、地址及排序，为尽快在线发表，请大家及时回复（我已认真校对，确认无误），我们收集到之后统一整理。谢谢

Dear participants, the cell death & disease articles submitted by us have been preliminarily accepted. As required by the journal, all participants need to confirm their name, email address and ranking. In order to publish online as soon as possible, please reply in time (I have carefully proofread and confirmed that it is correct), and we will sort it out after collecting it. thank you!

I have carefully proofread and confirmed that it is correct.

--

Department of Oncology, the First Affiliated Hospital of USTC, Division of Life Sciences and Medicine, University of Science and Technology of China, Hefei, Anhui Province, 230001, P.R.China.

6.Lingsuo Kong

回复：请确认自己的姓名、邮箱、地址及排序（投稿的Cell Death & Disease文章已初步接收） 发起会议 精简信息

发件人： 孔令锁 <konglingsuo3201@163.com>
时 间： 2021年11月09日 11:14:09 (星期二)
收件人： hdabing <hdabing@ustc.edu.cn>

I have carefully proofread and confirmed that it is correct, thank you!

发自 网易邮箱大师

---- 回复的原邮件 ----

发件人 Dabing Huang<hdabing@ustc.edu.cn>
日期 2021年11月09日 09:49
收件人 konglingsuo3201@163.com<konglingsuo3201@163.com>
主题 请确认自己的姓名、邮箱、地址及排序（投稿的Cell Death & Disease文章已初步接收）

各位参与人，我们投稿的Cell Death & Disease文章已初步接收。应期刊要求所有参与人需确认自己的姓名、邮箱、地址及排序，为尽快在线发表，请大家及时回复（我已认真校对，确认无误），我们收集到之后统一整理。谢谢

Dear participants, the cell death & disease articles submitted by us have been preliminarily accepted. As required by the journal, all participants need to confirm their name, email address and ranking. In order to publish online as soon as possible, please reply in time (I have carefully proofread and confirmed that it is correct), and we will sort it out after collecting it. thank you!

I have carefully proofread and confirmed that it is correct.

7.Hui Deng

Re:请确认自己的姓名、邮箱、地址及排序（投稿的Cell Death & Disease文章已初步接收） 发起会议 精简信息

发件人： hui <ahuideng@126.com>
时 间： 2021年11月09日 10:04:33 (星期二)
收件人： Dabing H... <hdabing@ustc.edu.cn>

I have carefully proofread and confirmed that it is correct. Thank you

在 2021-11-09 09:49:34，"Dabing Huang" <hdabing@ustc.edu.cn> 写道：

> 各位参与人，我们投稿的Cell Death & Disease文章已初步接收。应期刊要求所有参与人需确认自己的姓名、邮箱、地址及排序，为尽快在线发表，请大家及时回复（我已认真校对，确认无误），我们收集到之后统一整理。谢谢
>
> Dear participants, the cell death & disease articles submitted by us have been preliminarily accepted. As required by the journal, all participants need to confirm their name, email address and ranking. In order to publish online as soon as possible, please reply in time (I have carefully proofread and confirmed that it is correct), and we will sort it out after collecting it. thank you!

8.Fengmei Chao

回复：请确认自己的姓名、邮箱、地址及排序（投稿的Cell Death & Disease文章已初步接收） 发起会议 精简信息

发件人： 晁凤梅 <fengmei_chao@163.com>
时 间： 2021年11月09日 10:21:32 (星期二)
收件人： Dabing H... <hdabing@ustc.edu.cn>

I have carefully proofread and confirmed that it is correct, thank you!

晁凤梅
邮箱：fengmei_chao@163.com

签名由 网易邮箱大师 定制

在2021年11月09日 09:50，Dabing Huang 写道：

各位参与人，我们投稿的Cell Death & Disease文章已初步接收。应期刊要求所有参与人需确认自己的姓名、邮箱、地址及排序，为尽快在线发表，请大家及时回复（我已认真校对，确认无误），我们收集到之后统一整理。谢谢

Dear participants, the cell death & disease articles submitted by us have been preliminarily accepted. As required by the journal, all participants need to confirm their name, email address and ranking. In order to publish online as soon as possible, please reply in time (I have carefully proofread and confirmed that it is correct), and we will sort it out after collecting it. thank you!

I have carefully proofread and confirmed that it is correct.

--
Department of Oncology, the First Affiliated Hospital of USTC, Division of Life Sciences and Medicine, University of Science and Technology of China, Hefei, Anhui Province, 230001, P.R.China.

9.Ran Xia

回复：请确认自己的姓名、邮箱、地址及排序（投稿的Cell Death & Disease文章已初步接收） 发起会议 精简信息

发件人： 夏然 <xiar1108@163.com>

时 间： 2021年11月09日 11:13:49 (星期二)

收件人： hdabing <hdabing@ustc.edu.cn>

I have carefully proofread and confirmed that it is correct, thank you!

发自 网易邮箱大师

---- 回复的原邮件 ----

发件人 Dabing Huang<hdabing@ustc.edu.cn>

日期 2021年11月09日 09:50

收件人 xiar1108@163.com<xiar1108@163.com>

主题 请确认自己的姓名、邮箱、地址及排序（投稿的Cell Death & Disease文章已初步接收）

各位参与人，我们投稿的Cell Death & Disease文章已初步接收。应期刊要求所有参与人需确认自己的姓名、邮箱、地址及排序，为尽快在线发表，请大家及时回复（我已认真校对，确认无误），我们收集到之后统一整理。谢谢

Dear participants, the cell death & disease articles submitted by us have been preliminarily accepted. As required by the journal, all participants need to confirm their name, email address and ranking. In order to publish online as soon as possible, please reply in time (I have carefully proofread and confirmed that it is correct), and we will sort it out after collecting it. thank you!

10.Minghua Xie

回复：请确认自己的姓名、邮箱、地址及排序（投稿的Cell Death & Disease文章已初步接收） 发起会议 精简信息

发件人： xiemingh... <xieminghwa@163.com>

时 间： 2021年11月09日 16:13:43 (星期二)

收件人： Dabing H... <hdabing@ustc.edu.cn>

I have carefully proofread and confirmed that it is correct, thank you!

---原始邮件---

发件人: "Dabing Huang"<hdabing@ustc.edu.cn>

发送时间: 2021年11月9日(周二) 下午3:56

收件人: "xieminghwa"<xieminghwa@163.com>;

主题: 请确认自己的姓名、邮箱、地址及排序（投稿的Cell Death & Disease文章已初步接收）

各位参与人，我们投稿的Cell Death & Disease文章已初步接收。应期刊要求所有参与人需确认自己的姓名、邮箱、地址及排序，为尽快在线发表，请大家及时回复（我已认真校对，确认无误），我们收集到之后统一整理。谢谢

Dear participants, the cell death & disease articles submitted by us have been preliminarily accepted. As required by the journal, all participants need to confirm their name, email address and ranking. In order to publish online as soon as possible, please reply in time (I have carefully proofread and confirmed that it is correct), and we will sort it out after collecting it. thank you!

11.Fangfang Ge

Re:请确认自己的姓名、邮箱、地址及排序（投稿的Cell Death & Disease文章已初步接收） 发起会议 精简信息

发件人： gefangfa... <gefangfa...@163.com>

时 间： 2021年11月09日 17:59:31 (星期二)

收件人： Dabing H... <hdabing@ustc.edu.cn>

I have carefully proofread and confirmed that it is correct, thank you!

在 2021-11-09 15:57:19，"Dabing Huang" <hdabing@ustc.edu.cn> 写道：

> 各位参与人，我们投稿的Cell Death & Disease文章已初步接收。应期刊要求所有参与人需确认自己的姓名、邮箱、地址及排序，为尽快在线发表，请大家及时回复（我已认真校对，确认无误），我们收集到之后统一整理。谢谢
>
> Dear participants, the cell death & disease articles submitted by us have been preliminarily accepted. As required by the journal, all participants need to confirm their name, email address and ranking. In order to publish online as soon as possible, please reply in time (I have carefully proofread and confirmed that it is correct), and we will sort it out after collecting it. thank you!

12.Yueyin Pan

回复：请确认自己的姓名、邮箱、地址及排序（投稿的Cell Death & Disease文章已初步接收） 发起会议

精简信息

发件人： panyueyin <panyueyin@ustc.edu.cn>

时 间： 2021年11月09日 10:21:13 (星期二)

收件人： Dabing H... <hdabing@ustc.edu.cn>

I have carefully proofread and confirmed that it is correct, thank you!

panyueyin

邮箱：panyueyin@ustc.edu.cn

---- 回复的原邮件 ----

发件人 Dabing Huang<hdabing@ustc.edu.cn>

发送日期 2021年11月09日 09:51

收件人 panyueyin@ustc.edu.cn<panyueyin@ustc.edu.cn>

主题 请确认自己的姓名、邮箱、地址及排序（投稿的Cell Death & Disease文章已初步接收）

各位参与人，我们投稿的Cell Death & Disease文章已初步接收。应期刊要求所有参与人需确认自己的姓名、邮箱、地址及排序，为尽快在线发表，请大家及时回复（我已认真校对，确认无误），我们收集到之后统一整理。谢谢

Dear participants, the cell death & disease articles submitted by us have been preliminarily accepted. As required by the journal, all participants need to confirm their name, email address and ranking. In order to publish online as soon as possible, please reply in time (I have carefully proofread and confirmed that it is correct), and we will sort it out after collecting it. thank you!

13.Shanbao Cai

Re: 请确认自己的姓名、邮箱、地址及排序（投稿的Cell Death & Disease文章已初步接收） 发起会议

发件人： sbcsbc

时 间： 2021年11月10日 11:20:04 (星期三)

收件人： Dabing H... <hdabing@ustc.edu.cn> puyouguang <puyouguang@163.com> puyouguang <puyouguang@126.com>

I have carefully proofread and confirmed that it is correct.

-----Original Messages-----
From:"Dabing Huang" <hdabing@ustc.edu.cn>
Sent Time:2021-11-09 09:51:57 (Tuesday)
To: sbcsbc@ustc.edu.cn
Cc:
Subject: 请确认自己的姓名、邮箱、地址及排序（投稿的Cell Death & Disease文章已初步接收）

各位参与人，我们投稿的Cell Death & Disease文章已初步接收。应期刊要求所有参与人需确认自己的姓名、邮箱、地址及排序，为尽快在线发表，请大家及时回复（我已认真校对，确认无误），我们收集到之后统一整理。谢谢

Dear participants, the cell death & disease articles submitted by us have been preliminarily accepted. As required by the journal, all participants need to confirm their name, email address and ranking. In order to publish online as soon as possible, please rep
proofread and confirmed that it is correct), and we will sort it out after collecting it. thank you!
