# Supplementary material for: LAMTOR5-AS1 regulates chemotherapy-induced oxidative stress by controlling the expression level and transcriptional activity of NRF2 in osteosarcoma cells
Source: Cell Death Dis. 2021 Dec 3;12(12):1125. doi: 10.1038/s41419-021-04413-0 (PMC8642434; doi:10.1038/s41419-021-04413-0)
Supplement: Supplementary file 2 — Supplementary materials [file 41419_2021_4413_MOESM2_ESM.pdf]

Supplementary Information for

LAMTOR5-AS1 regulates the chemotherapy-induced oxidative stress via controlling the expression level and transcriptional activity of NRF2 in osteosarcoma cells

Supplementary Information

Supplementary materials and methods

RNA extraction and real-time PCR analyses

Total RNA was extracted from cultured cells with Trizol reagent (Tiangen, China). For real-time PCR, RNA was retrieved to the cDNA using a reverse transcription kit (Takara, Japan). In addition, the RNA levels of LAMTOR5-AS1, NRF2, and KEAP1 genes were quantified by real-time PCR analysis, and the TaqMan probes with different fluorescence intensity were used in the FTC-3000p PCR instrument (Funglyn, China). The level of beta-actin was normalized by $2^{-\Delta\Delta Ct}$ before comparing the relative levels of target genes.

Cell transfection assays

Using riboFECT CP transfection kit provided by Guangzhou Ribobio, China, smart silencer (ssRNA), siRNA or DNA plasmid transfection was carried out on 6-well plate. In the functional analysis, 100 nM ss-LAMTOR5-AS1 or si-LAMTOR5-AS1, si-NRF2 or si-KEAP1 were introduced into the cells in the culture medium, and then harvested for further detection. The smart silencers of LAMTOR5-AS1 (Ribo^{TM} lncRNA smart silencer can inhibit lncRNA in cell nucleus and cytoplasm simultaneously), the siRNAs of LAMTOR5-AS1, NRF2 and KEAP1 were purchased from RiboBio (China), Detailed information regarding the siRNA sequences were depicted in this appendix Table S4.

Drug resistance profiling (IC_{50} measurements) assays

Three clinical grade chemotherapeutic drugs were: VP-16 (etoposide, Hengrui, China), CBP (carboplatin, Qilu, China) and DDP (cisplatin, Haosen, China). Cells in logarithmic growth phase were inoculated into 96-well plate three times at the density of about 1.0×10^4/hole, after attached, treated with the drug concentration of IC_{50} for 72 hours, observed the cell death once a day. Cell viability was measured using cell counting kit 8 (CCK-8) (Beyotime, China) with the optical density of 450-nm (Tecan, Switzerland).

Cell apoptosis assays

Infected or transfected cells were harvested and rinsed twice with pre-cooling PBS. The samples were diluted with 150 µl of 1×annexin-binding buffer, then 5 µl of FITC-labeled enhanced annexinV and 5 µl (20 µg/ml) of propidium iodide (PI, Beyotime, China) were added. Then the cells were incubated in the dark for 15 minutes at room temperature. Flow cytometry was conducted on a FACSCalibur instrument (BD, America).

Cell proliferation assay and colony formation assays

Cell proliferation was measured using the CCK-8 according to the manufacturer's instructions, checked every 24 hours (0, 24, 48, 72 and 96 hours). For the colony formation assay, about 500-1,000 infected or transfected cells were seeded into each well of a 6-well plate and maintained in a medium containing 10% FBS for 10 days. The colonies were fixed with methanol and stained with 0.1% crystal violet, and the number of clones was counted.

5-Ethynyl-2′-deoxyuridine (EDU) assays

Proliferation of osteosarcoma cells were also monitored using BeyoClick™ EdU Cell Proliferation Kit with Alexa Fluor 488 (Beyotime, China) according to the manufacturer's instructions. G-292 cell was seeded in 96-well plates and transfected with ss-LAMTOR5-AS1 or the negative control. After 24 hours, cells were treated with IC_{50}

concentration of DDP for 48 hours and stained with 50 μM EdU for 2 hours. SJSA-1 infected with LAMTOR5-AS1-OE were directly treated with IC_{50} concentration of DDP for 48 hours and stained with 50 μM EdU for 2 hours. All the cells nuclei were stained with DAPI for 1 hour, then the cells were then examined using a florescence microscope (Olympus, Japan).

TdT-mediated dUTP Nick-End Labeling (TUNEL) assays

The TUNEL assay was performed using One Step TUNEL Apoptosis Assay Kit (Beyotime, China) to label 3′-end of fragmented DNA of the apoptotic cells. In brief, G-292 cell was seeded in 96-well plates and transfected with ss-LAMTOR5-AS1 or the negative control. After 24 hours, cells were treated with IC_{50} concentration of DDP for 48 hours. SJSA-1 infected with LAMTOR5-AS1-OE were directly treated with IC_{50} concentration of DDP for 48 hours.Then cells were treated as indicated were fixed with 4% paraform phosphate buffer saline, rinsed with PBS, then permeabilized by 0.1% Triton X-100 for FITC end-labeling the fragmented DNA of the apoptotic cells using TUNEL cell apoptosis detection kit. The FITC-labeled TUNEL-positive cells were imaged under a fluorescent microscopy by using 488-nm excitation and 530-nm emission.

Supplementary Tables:

Table S1. List of lncRNAs upregulated in G-292 than SJSA-1 cell lines

Gene ID	Name	Position	Strand	SJSA-1 RPKM	G-292 RPKM	G-292/SJSA-1
ENST00000582491	CRHR1-IT1	chr17:45638975-45646229	+	0.008	0.135	17.290
ENST00000582847	TTN-AS1	chr2:178522827-178620217	+	0.052	0.824	15.787
ENST00000354453	LAT	chr16:28984826-28990783	+	0.763	9.807	12.858
ENST00000432518	LINC00693	chr3:28575278-28758337	+	0.018	0.202	11.527
ENST00000397787	COL18A1-AS1	chr21:45419716-45425070	-	0.021	0.227	10.835
ENST00000414786	ANK3	chr10:60026298-60733526	-	0.024	0.250	10.486
ENST00000602385	TERC	chr3:169764610-169765060	-	164.017	1033.610	6.302
ENST00000439819	DICER1-AS1	chr14:95157688-95179933	+	0.289	1.479	5.115
ENST00000443196	UPK1A-AS1	chr19:35667948-35673291	-	0.164	0.799	4.867
ENST00000502049	LRP4-AS1	chr11:46846411-46874416	+	0.217	0.890	4.110
ENST00000328404	TEX40	chr11:64300391-64304767	+	0.253	0.966	3.813
ENST00000515356	ZBED3-AS1	chr5:77086798-77148351	+	0.089	0.337	3.787
ENST00000441531	ACVR2B-AS1	chr3:38451027-38454820	-	0.144	0.512	3.550
ENST00000392630	C10orf91	chr10:132445210-132448321	+	0.039	0.136	3.458
ENST00000608990	C20orf203	chr20:32631625-32651981	-	0.006	0.021	3.458
ENST00000460833	ADAMTS9-AS2	chr3:64684870-65011468	+	0.052	0.180	3.458
ENST00000598158	LAMTOR5-AS1	chr1:110407809-110416274	+	0.136	0.471	3.458
ENST00000502764	HOXB-AS1	chr17:48544351-48551241	+	0.637	1.951	3.063
ENST00000420193	PRKG1-AS1	chr10:52296848-52314128	-	0.032	0.096	2.997
ENST00000421378	LINC00271	chr6:135497801-135690838	+	0.035	0.102	2.882
ENST00000505495	CACNA1G-AS1	chr17:50556207-50562108	-	0.030	0.082	2.690
ENST00000414282	SNHG7	chr9:136724594-136728184	-	102.005	258.103	2.530
ENST00000421598	BSN-AS2	chr3:49549306-49554366	-	0.033	0.084	2.497

ENST00000449175	MIR600HG	chr9:123109494-123115477	-	0.269	0.671	2.492
ENST00000437615	LINC00242	chr6:169788790-169798825	-	0.012	0.028	2.305
ENST00000429328	LINC00853	chr1:47179250-47180339	+	0.088	0.202	2.305
ENST00000423546	FAM225B	chr9:113104723-113111677	-	0.007	0.017	2.305
ENST00000461286	RFPL1S	chr22:29437015-29442129	-	0.003	0.007	2.305
ENST00000414532	MATN1-AS1	chr1:30718772-30726746	+	0.524	1.207	2.305
ENST00000435328	RAPGEF4-AS1	chr2:172723189-172736206	-	0.013	0.025	2.017

Table S2. List of qPCR primers

**LAMTOR5-AS1 probe**	**Sequence**
hLAMTOR5-AS1 F	GCCTCAGTGCTTCAGTTCGTG
hLAMTOR5-AS1 R	CTTTCCCAAAAGACATCAAGACTC
hLAMTOR5-AS1 P	TCATCTCTGCATCCCTAGTGCAACAGAG
**NRF2(NFE2L2) probe**	**Sequence**
hNRF2 F	CCACATTCCCAAATCAGATGC
hNRF2 R	CTCGATGTGACCGGGAATATC
hNRF2 P	ROX-CATGCAGCTTTTGGCGCAGACATTC-BHQ2
**Biotin RNA pull-down**	**Sequence**
LAMTOR5-AS1-sense-Biotin-1	CCCCGCGCGGTGACCGTCGAGGTGACCTGC
LAMTOR5-AS1-sense-Biotin-2	GCTTAGAGTTCTGCACCCCCCAGCATGGAG
LAMTOR5-AS1-sense-Biotin-3	CCCCCACTATAAATGGGAAGATAAATTCAC
LAMTOR5-AS1-antisense-Biotin-1	GCAGGTCACCTCGACGGTCACCGCGCGGGG
LAMTOR5-AS1-antisense-Biotin-2	CTCCATGCTGGGGGGTGCAGAACTCTAAGC
LAMTOR5-AS1-antisense-Biotin-3	GTGAATTTATCTTCCCATTTATAGTGGGGG
LAMTOR5-AS1-antisense-Biotin(1-150)	GCAGGTCACCTCGACGGTCACCGCGCGGGG
LAMTOR5-AS1-sense(1-150)	CCCCGCGCGGTGACCGTCGAGGTGACCTGC
LAMTOR5-AS1-antisense-Biotin(151-300)	GAGTAAAGGTAACCATAGAGTTCCTTTCCC
LAMTOR5-AS1-sense(151-300)	GGGAAAGGAACTCTATGGTTACCTTTACTC
LAMTOR5-AS1-antisense-Biotin(301-450)	CTCCATGCTGGGGGGTGCAGAACTCTAAGC
LAMTOR5-AS1-sense(301-450)	GCTTAGAGTTCTGCACCCCCCAGCATGGAG
LAMTOR5-AS1-antisense-Biotin(451-533)	GTGAATTTATCTTCCCATTTATAGTGGGGG
LAMTOR5-AS1-sense(451-533)	CCCCCACTATAAATGGGAAGATAAATTCAC
**Co-immunoprecipitation**	**Sequence**
Flag-KEAP1 sense	GTGCGGCCGCAATGCAGCCAGATCCCAGGCC
Flag-KEAP1 antisense	GCGTCGACTCAACAGGTACAGTTCTGCT
GFP-P62 sense	CGGAATTCCATGGCGTCGCTCACCGTGAAGGCCT
GFP-P62 antisense	CGGTCGACTCACAACGGCGGGGGATGCTTTGAA
HA-NRF2 sense	CGCTCGAGACATGATGGACTTGGAGCTGCCGCCGC
HA-NRF2 antisense	CGGGATCCCTAAAGGTCCTCCTCGGAGATAAGC
LAMTOR5-AS1 sense	GAGTCTTGATGTCTTTTGGGAAAGG
LAMTOR5-AS1 antisense	TCCAAGGGGAATGTGGGAGT
KEAP1 sense	ACGGGACAAACCGCCTTAAT

KEAP1 antisense	ATACAGTTGTGCAGGACGCA	
NRF2 sense	CAACTACTCCCAGGTTGCCC	
NRF2 antisense	AGTGACTGAAACGTAGCCGAA	
**ARE plasmid construction**	**Sequence**	
LAMTOR5-AS1 sense	GAGTCTTGATGTCTTTTGGGAAAGG	
LAMTOR5-AS1 antisense	TCCAAGGGGAATGTGGGAGT	
KEAP1 sense	ACGGGACAAACCGCCTTAAT	
KEAP1 antisense	ATACAGTTGTGCAGGACGCA	
NRF2 sense	CAACTACTCCCAGGTTGCCC	
NRF2 antisense	AGTGACTGAAACGTAGCCGAA	
**Chromatin immunoprecipitation**	**Sense**	**Antisense**
LAMTOR5-AS1	TCACGTGTCTTCCAAGTGCTG	GGTTCTGAGCCGGAGTGGTC
LAMTOR5-AS1-BS1	TTGAACTCCTGGGCTCAAGT	GCCCTCATTTTCTTCACTGG
LAMTOR5-AS1-BS2	TCAAATAACAACCCAGTGAAGAAA	CACAACAACCCGGCAAG
HMOX1	ACTGGCATCCTGCTTTATGTG	ACAACTGACCTGTGAGGGTAGAT
**RNA immunoprecipitation**	**Sense**	**Antisense**
LAMTOR5-AS1	GAGTCTTGATGTCTTTTGGGAAAGG	TCCAAGGGGAATGTGGGAGT
**Fluorescence *in situ* hybridization**	**Sequence**	**Modified**
LAMTOR5-AS1 probe1	CATCAGAGGATTTGTCCACAGGCTTAC	5'CY3
LAMTOR5-AS1 probe2	CATCAGAGGATTTGTCCACAGGCTTAC	5'CY3
LAMTOR5-AS1 probe3	GGGGAAAACCGAGGTATCAAGGC	5'CY3
NRF2 probe	CGTCTAAATCAACAGGGGCTAC	5'CY3
NRF2 probe1	TAAAGTAGCAGGTGAGGGCATG	5'CY3
NRF2 probe2	CGAGATATAAGGTGCTGAGTTG	5'CY3
KEAP1 probe	CTGTCGATCTGGTACATGACAG	5'CY3
KEAP1 probe1	CTCGAAGATCTTGACCAGGTAG	5'CY3
KEAP1 probe2	CAGGAACGTGTGACCATCATAG	5'CY3
18S	CTGCCTTCCTTGGATGTGGTAGCCGTTTC	5'CY3
NC	TGCTTTGCACGGTAACGCCTGTTTT	5'CY3

Table S3. List of antibodies

Antibody	Catalogue NO.	Company
NRF2, NFE2L2 Antibody	16396-1-AP	Proteintech, USA
KEAP1 Antibody	10503-2-AP	Proteintech, USA
P62/SQSTM1 Antibody	18420-1-AP	Proteintech, USA
anti-GSK3 alpha / beta phospho (Tyr216) antibody	ARG51777	Arigo, China
GSK3B Antibody	22104-1-AP	Proteintech, USA
AKT3 Polyclonal Antibody	YM3251	ImmunoWay, China
AKT3 Antibody	21641-1-AP	Proteintech, USA
AKT-Phospho-S473 Antibody	66444-1-Ig	Proteintech, USA
AKT1 Antibody	10176-2-AP	Proteintech, USA
HMOX1 Antibody	10701-1-AP	Proteintech, USA

Thioredoxin Antibody	14999-1-AP	Proteintech, USA

Table S4. siRNA and ssRNA sequences

**si-LAMTOR5-AS1**	**sense**	**antisense**
si-LAMTOR5-AS1-homo-170	CCCUAGUGCAACAGAGCAUTT	AUGCUCUGUUGCACUAGGGTT
si-LAMTOR5-AS1-homo-235	GGAAAGGAACUCUAUGGUUTT	AACCAUAGAGUUCCUUUCCTT
si-LAMTOR5-AS1-homo-361	GCAGAGCGCAAAGAUUUCUTT	AGAAAUCUUUGCGCUCUGCTT
si-LAMTOR5-AS1-homo-452	GCCUGUGGACAAAUCCUCUTT	AGAGGAUUUGUCCACAGGCTT
**ss-LAMTOR5-AS1**	ASO sequence	siRNA sequence
	AGTGCAACAGAGCATGACAA	ATCCTCTGATGCCTTGATA
	CTCAGTGCTTCAGTTCGTGG	CTTGTAAGCCTGTGGACAA
	TGACCTGCACCTGGCTCCAT	CTGGTCTACTGTCACAACA
**si-NRF2**	**sense**	**antisense**
NRF2-homo-1118	GGGAGGAGCUAUUAUCCAUTT	AUGGAUAAUAGCUCCUCCCTT
NRF2-homo-1500	GCCCAUUGAUGUUUCUGAUTT	AUCAGAAACAUCAAUGGGCTT
NRF2-homo-1845	GCCUGUAAGUCCUGGUCAUTT	AUGACCAGGACUUACAGGCTT
**si-KEAP1**	**sense**	**antisense**
KEAP1-homo-771	CCCGGGAGUACAUCUACAAUTT	AUGUAGAUGUACUCCCGGGTT
KEAP1-homo-1053	GCAAGGACUACCUGGUCAATT	UUGACCAGGUAGUCCUUGCTT
KEAP1-homo-1906	GGAGUGUUACGACCCAGAUTT	AUCUGGGUCGUAACACUCCTT

Table S5. List of reagents

**Reagents**	**Company**
VP-16 (etoposide)	Hengrui, China
CBP (carboplatin)	Qilu, China
DDP (cisplatin)	Haosen, China
CHX (cycloheximide)	Merck, Germany
Mg132 (proteasome inhibitor)	Merck, Germany
CCK-8 (cell counting Kit-8)	Bimake, China
PI (propidium iodide)	Beyotime, China

Supplementary Figures:

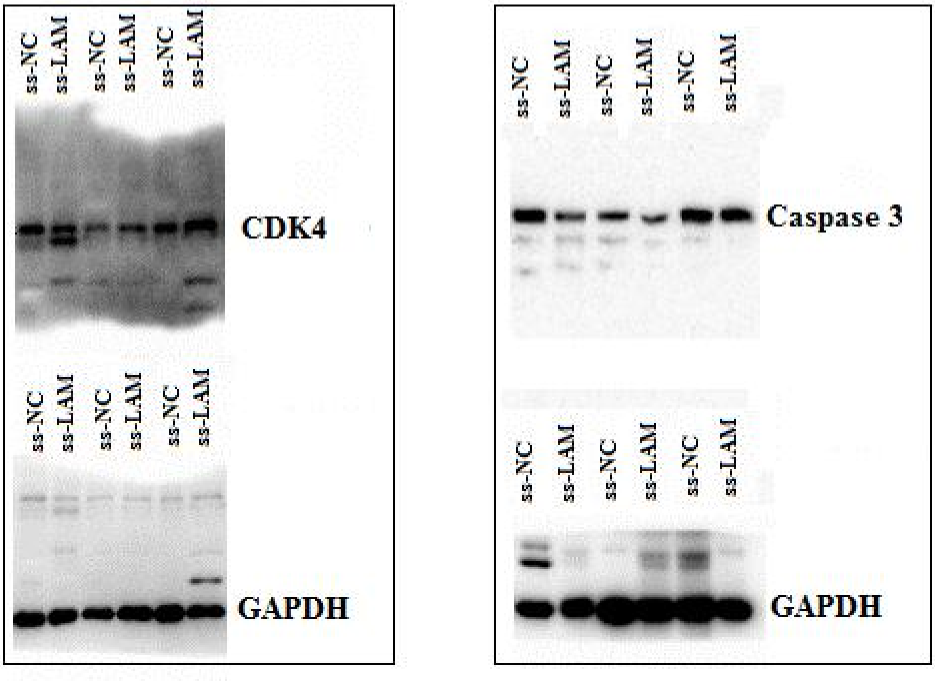

Fig S1. The expression of apoptotic related proteins Caspase 3 and CDK4 in G-292-derived xenografts.

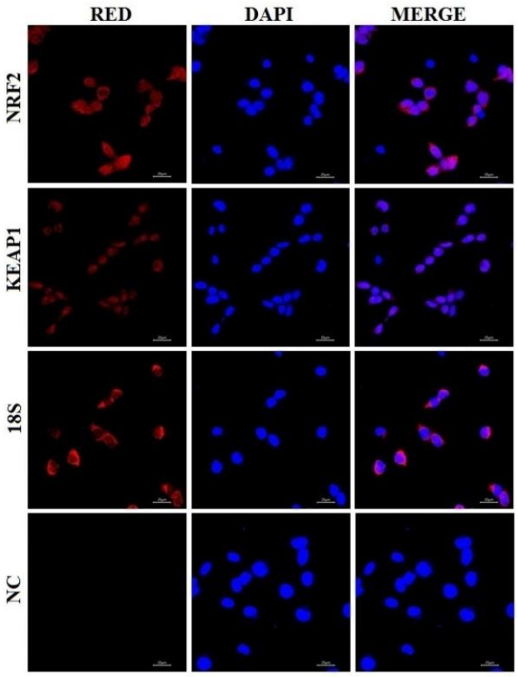

Fig S2. The the localization of NRF2 and KEAP1 in HEK293T cells.

RNA fluorescence in situ hybridization showing the localization of NRF2 and KEAP1 in HEK-293T cells. Cells were incubated with three different sense probes. After DAPI staining, fluorescence was observed under a fluorescence microscope. Scale bar, 25μm. NC FISH probe and 18S FISH probe were used as negative and cytoplasm control respectively. The experiments were performed independently three times.

Figure 3B

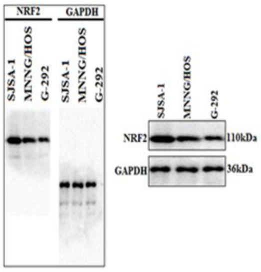

Figure 3C

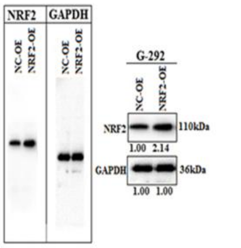

Figure 3D

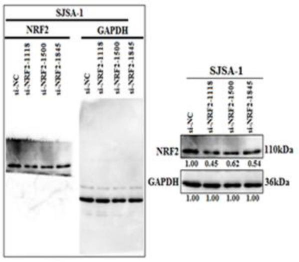

Figure 3E

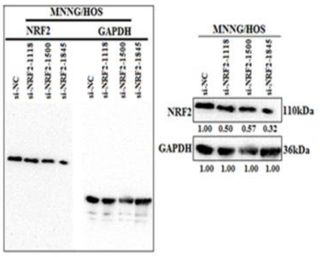

Fig S3. The full-length gels of the Figure3B, Figure3C, Figure3D and Figure3E, western analyses used in the revised manuscript.

Figure 4D

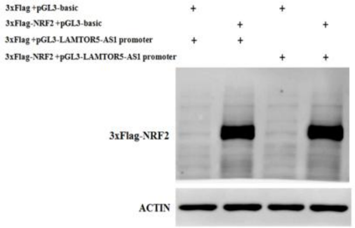

Figure 4F

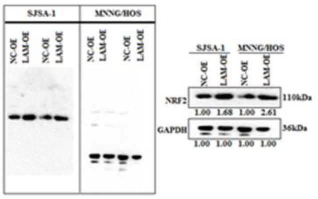

Figure 4G

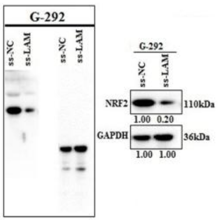

Figure 4I

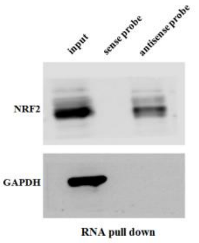

Figure 4J

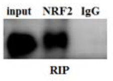

Figure 4K

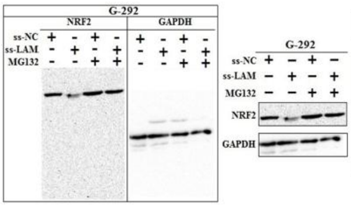

Figure 4L

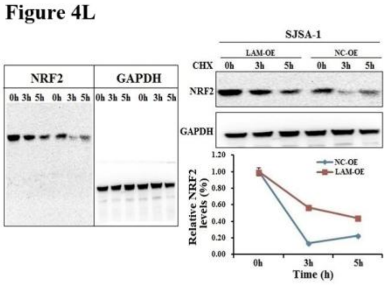

Fig S4. The full-length gels of the Figure4D, Figure4F, Figure4G, Figure4I, Figure4J, Figure4K, and Figure4L,western analyses used in the revised manuscript.

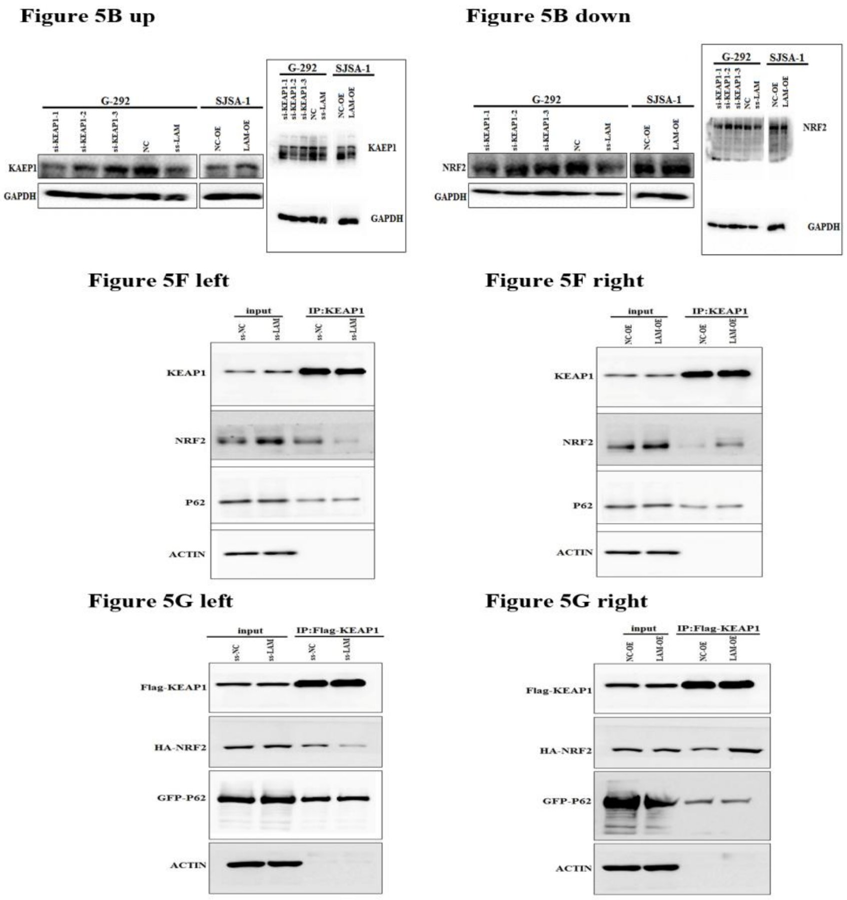

Fig S5. The full-length gels of the Figure5B, Figure5F and Figure5G,western analyses used in the revised manuscript.

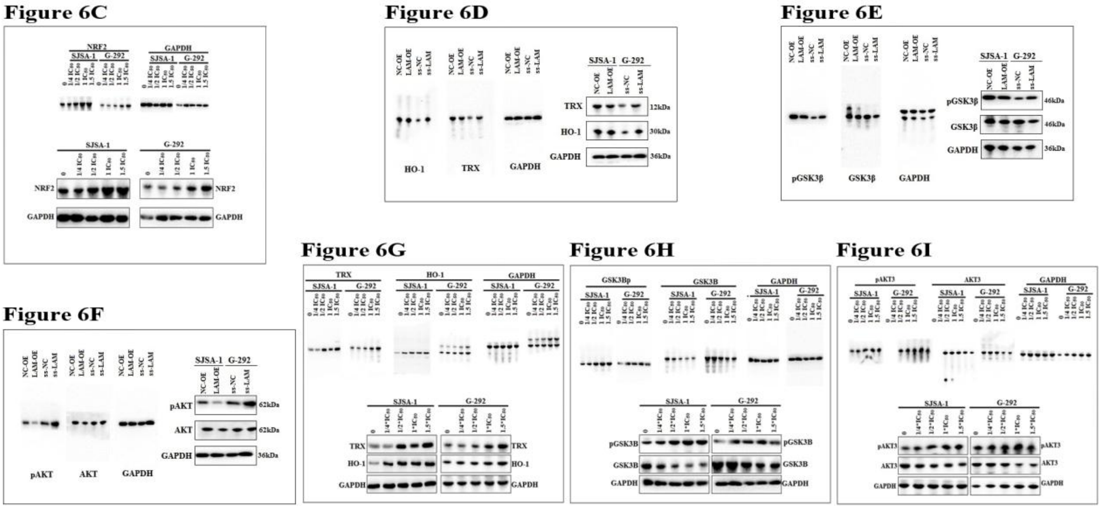

Fig S6. The full-length gels of the Figure6C-6I,western analyses used in the revised manuscript.

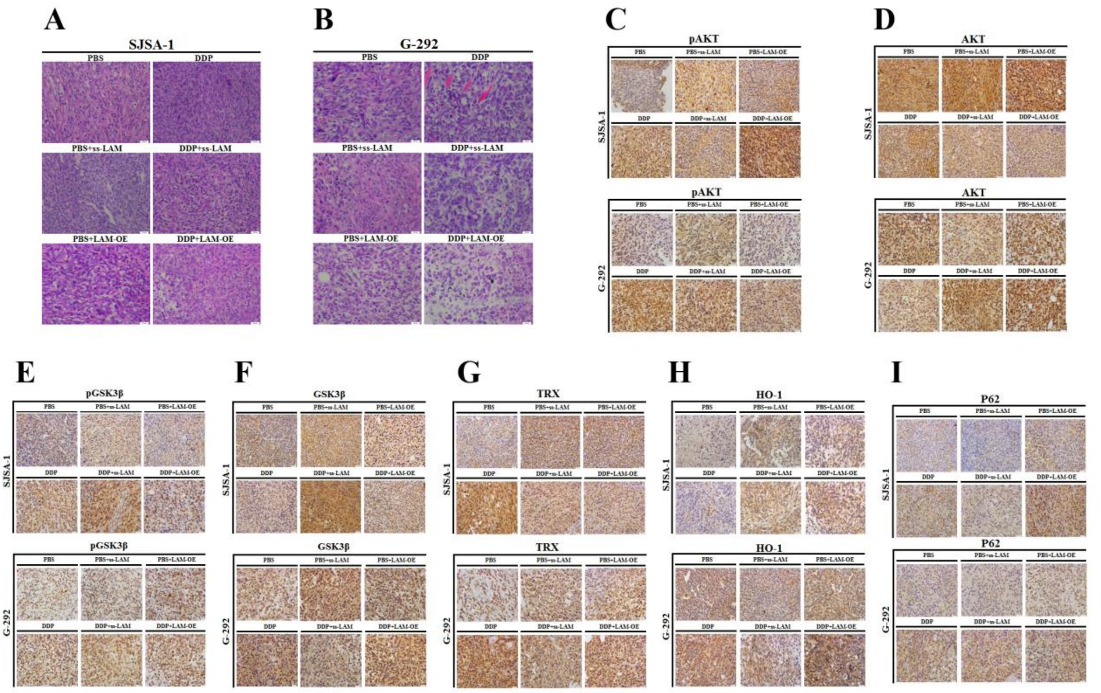

Fig S7. The effect of LAMTOR5-AS1 on the *in vivo* growth and DDP drug resistance of SJSA-1 and G-292-derived xenografts in nude mice.

(A and B): The hematoxylin & eosin (H&E) staining assays on tumors of SJSA-1-derived xenograft model. Representative images from the LAMTOR5-AS1 (ss-LAMTOR5-AS1 and LAMTOR5-AS1-OE), IC_{50} dose of DDP and control groups.

(C-I): The protein levels of phosphorylated AKT3, AKT3, phosphorylated GSK3β, GSK3β, TRX, HO-1 and P62 in each group were determined by Immunohistochemistry (Magnification: 200×).

Supplementary Sequences:

Sequence S1.

Homo sapiens LAMTOR5 antisense RNA 1 (LAMTOR5-AS1), long non-coding RNA NCBI Reference Sequence: NR_102697.1

GACGAAGGCTTGGGCTCCCCGCGCGGTGACCGTCGAGGTGACCTGCACCTGGCTCCATGGCGGAACCGGCCGGCACGGATTATTCCCCTCTCGG

GAGAGGTCTGCAGCCGGGCCTCAGTGCTTCAGTTCGTGGCCTGGCATTTTGTGAAGAGTGTCTTCATCTCTGCATCCCTAGTGCAACAGAGCATG

ACAAAAATTCAACAAATATTTATTGAAGAGTCTTGATGTCTTTTGGGAAAGGAACTCTATGGTTACCTTTACTCTAAGATTAGATGGGTGTTACTG

ATTCTTACCTCAGAGAAGAGAATGTCATGGAGACAAAATGACCTGCTTAGAGTTCTGCACCCCCCAGCATGGAGAGCAGAGCGCAAAGATTTCT

TCCGGACTCCCACATTCCCCTTGGAATCCAAGAACTGGTCTACTGTCACAACACTTTAGTACCCTCTTGTAAGCCTGTGGACAAATCCTCTGATG

CCTTGATACCTCGGTTTTCCCCCACTATAAATGGGAAGATAAATTCACTAGAAACAGTA

Sequence S2.

LAMTOR5-AS1 promoter (the underline part indicates the binding sites of Nrf2)

AAAACAACAACAACAACAACAAAAACACAAAACCCTGAGGCTCAGAAGATAAATATTTGCTGAAGATCACTGTGCTTGTAAGTGGTGGAGCC

TGACCATTAACTTCCCCACGACACTGCTTCAATTTCTATCACTTCATCAACCACACAACCCGAGAACTGTGAGGGGATTTAGCAGGCCCAGTCC

AGATCTCTTATTGTACAGAGAAGAAATCTGAGGCTCAGAGAGGGGAAGGGACTAATTTGGACCTTTAACTTCGGATTCTTAACTTTCTTTCTGG

TGTTCTAACTATACTGTACTACAAGAGGCTGTCTTAGAGCTTTAGTTCAACCAGCTTCAGAGATACTCATTGTTATGGGTTGAATTATGTCTCCC
CAAAATTCATATGTCAAAGTTCTAACCCCCAGTACCTCAGAATGTGACCATATTTGGAGATAGGACCTTTACAGAGGTAATCAGGTGAAAATG
AGGTTGTTAGGGTGGGCCTTAGTCCAGTATGGCTGGTGTCCTCATAAAAAGGGGAAATTTGGACACAGATGTACATAGAGGGAAGATGGTGTG
AAGAGACATAAAGAACAGATAGTCATCTATAGCCAAGGCCTGGAACAGATCCTTCCCTAACAGCCCTCAGAAGGAAGCAACCTCACTGACAC
CTTGATTTTGGACTGCTACCCTCTGGCACTGTGAGACAATAAATTCATGTTGGTTAGGCCACCCAGTTTGTTATGGCAGCCCTAGGAAACCTTT
GTGGATATATCTGTTCGTATACCCATAAAGTCTGCAAGTCAAAAATATCACAGACTTTTGGCAGCCCTTCATTCAAGCACTGGCTGGGAGGGTC
ACCCTCCCCGAGGCAAAAGGATAGAGCAAATGATCTTTCCAGCTTGCTCCCAACCTTGATCTTTGGCCAAGATATTTTTCTTTCACTGGACATT
GGCCAACATTGGTGTCATAACTTAATTTGACTGTGACCTTCTTGAGTCTTTTATGTCTCTGAGCTTGACCTGTAGAAGAAGCTCAATAAATGTG
GGTAGAGGGGATGAGTGGGAAAATCAGCTCCCAGCCATTCCAGTTCAAGCCTCTTCAAGCAGAAGAGTCCCACCTGAAAGACCTCATTTTCAC
TTGATTACTCGATCTCCAAGCAATAGGTGGGGCAGGAAGCAGGGTATGTAAGGCCTGTCAGTGGATGGAAGTTGAAGTTGGACACCATCTCCT
AAAGTTTAGAGCTGAGCCCAAATTCTGGAAAACACAAGCAAAATACATTTTATATGACTCAGGATGGGCTAAAGAAGTTCCAGTACAGGAAA
CTGTTTTCCTGCTGCTTTATTAATTTTGCTACAGAGGATAAATGAGATGCAGACTTTCATATCCAAGTCTTCATTAGATGCCCAGTTAATGCAGC
CATGACAAATGGGAATGAAGCCCTTATGAATAAAAAAAAGATCAACCCAGTATAAAAAAATCATAGACCAGAGAAATGAAAATTTAGAGGA
GCTGATAAAATACCCTATCCTTAATTAGACTGTCAGCATACACATCAAATCAGCCTTAATGCCAAGATTTATTGGCCTTGGAATTGGGTAAATG
AAATAAGTGCTTACAAATGAATGGAAAAGAAATTACCTGTATTAAAACGTATTTGTGATGCAGGCCTGTGCACTGAGGGTTTTAGTCAAACTC
AGGCTTGCAGCTTCTTTTGCTCTGCATCCATTTCCACCCCATGTTCCCCAGTGGGTTTCAGAGACCCTCTTTGTGGGGTAACAATATGTACCATT
GTCAACTACCTCTGCTGTTGTCCTGGAAAAATCCTTCTTGGACAGTAAAATATTTTTGTCTTTATTTCACCATCTCCAGATTAGATGGGTGTTAC
TGATTCTTACCTCAGAGAAGAGAATGTCATGGAGACAAAATGACCTGCTTAGAGTTCTGCACCCCCCAGCATGGAGAGCAGAGCGCAAAGATT
TCTTCCGGACTCCCACATTCCCCTTGGAATCCAAGAA

Sequence S3.

NFE2L2 (NRF2) gene ID 4780 Homo sapiens (human)

ATGATGGACTTGGAGCTGCCGCCGCCGGGACTCCCGTCCCAGCAGGACATGGATTTGATTGACATACTTTGGAGGCAAGATATAGATCTTGGAGT
AAGTCGAGAAGTATTTGACTTCAGTCAGCGACGGAAAGAGTATGAGCTGGAAAAACAGAAAAAACTTGAAAAGGAAAGACAAGAACAACTCC
AAAAGGAGCAAGAGAAAGCCTTTTTCGCTCAGTTACAACTAGATGAAGAGACAGGTGAATTTCTCCCAATTCAGCCAGCCCAGCACATCCAGTC
AGAAACCAGTGGATCTGCCAACTACTCCCAGGTTGCCCACATTCCCAAATCAGATGCTTTGTACTTTGATGACTGCATGCAGCTTTTGGCGCAGA
CATTCCCGTTTGTAGATGACAATGAGGTTTCTTCGGCTACGTTTCAGTCACTTGTTCCTGATATTCCCGGTCACATCGAGAGCCCAGTCTTCATTG
CTACTAATCAGGCTCAGTCACCTGAAACTTCTGTTGCTCAGGTAGCCCCTGTTGATTTAGACGGTATGCAACAGGACATTGAGCAAGTTTGGGAG
GAGCTATTATCCATTCCTGAGTTACAGTGTCTTAATATTGAAAATGACAAGCTGGTTGAGACTACCATGGTTCCAAGTCCAGAAGCCAAACTGAC
AGAAGTTGACAATTATCATTTTTACTCATCTATACCCTCAATGGAAAAAGAAGTAGGTAACTGTAGTCCACATTTTCTTAATGCTTTTGAGGATTCC
TTCAGCAGCATCCTCTCCACAGAAGACCCCAACCAGTTGACAGTGAACTCATTAAATTCAGATGCCACAGTCAACACAGATTTTGGTGATGAATT
TTATTCTGCTTTCATAGCTGAGCCCAGTATCAGCAACAGCATGCCCTCACCTGCTACTTTAAGCCATTCACTCTCTGAACTTCTAAATGGGCCCATT
GATGTTTCTGATCTATCACTTTGCAAAGCTTTCAACCAAAACCACCCTGAAAGCACAGCAGAATTCAATGATTCTGACTCCGGCATTTCACTAAA
CACAAGTCCCAGTGTGGCATCACCAGAACACTCAGTGGAATCTTCCAGCTATGGAGACACACTACTTGGCCTCAGTGATTCTGAAGTGGAAGAG
CTAGATAGTGCCCCTGGAAGTGTCAAACAGAATGGTCCTAAAACACCAGTACATTCTTCTGGGGATATGGTACAACCCTTGTCACCATCTCAGGG
GCAGAGCACTCACGTGCATGATGCCCAATGTGAGAACACACCAGAGAAAGAATTGCCTGTAAGTCCTGGTCATCGGAAAACCCCATTCACAAA
AGACAAACATTCAAGCCGCTTGGAGGCTCATCTCACAAGAGATGAACTTAGGGCAAAAGCTCTCCATATCCCATTCCCTGTAGAAAAAATCATTA
ACCTCCCTGTTGTTGACTTCAACGAAATGATGTCCAAAGAGCAGTTCAATGAAGCTCAACTTGCATTAATTCGGGATATACGTAGGAGGGGTAAG
AATAAAGTGGCTGCTCAGAATTGCAGAAAAAGAAAACTGGAAAATATAGTAGAACTAGAGCAAGATTTAGATCATTTGAAAGATGAAAAAGAA
AAATTGCTCAAAGAAAAAGGAGAAAATGACAAAAGCCTTCACCTACTGAAAAAACAACTCAGCACCTTATATCTCGAAGTTTTCAGCATGCTAC
GTGATGAAGATGGAAAACCTTATTCTCCTAGTGAATACTCCCTGCAGCAAACAAGAGATGGCAATGTTTTCCTTGTTCCCAAAAGTAAGAAGCCA
GATGTTAAGAAAAACTAG

Sequence S4.

ARE promoter-wt (the underline part indicates the binding sites of NRF2)

GAGCTC<u>CGCGTGACTCAGCA</u>TCTAGT<u>CGCGTGACTCAGCA</u>TCTAGTTGTCACGTCCTGCACGACGCTAGCGAGATCCGGCCCCGCCCAGCGTCT

TGTCATTGGCGAATTCGAACACGCAGATGCAGTCGGGGCGGCGCGGTCCGAGGTCCACTTCGCATATTAAGGTGACGCGTGTGGCCTCGAACAC

CGAGCGACCCTGCAGCGACCCGCTTAACAGCGTCAACAGCGTGCCGCAGATCTAAGTAAGCTTGGCATTCCGGTACTGTTGGTAA

ARE promoter-mut

GAGCTCTCTAGTTCTAGTTGTCACGTCCTGCACGACGCTAGCGAGATCCGGCCCCGCCCAGCGTCTTGTCATTGGCGAATTCGAACACGCAGATG

CAGTCGGGGCGGCGCGGTCCGAGGTCCACTTCGCATATTAAGGTGACGCGTGTGGCCTCGAACACCGAGCGACCCTGCAGCGACCCGCTTAAC

AGCGTCAACAGCGTGCCGCAGATCTAAGTAAGCTTGGCATTCCGGTACTGTTGGTAA
